# Tailored theranostic apolipoprotein E3 porphyrin-lipid nanoparticles target glioblastoma†
†Electronic supplementary information (ESI) available. See DOI: 10.1039/c7sc00732a



**DOI:** 10.1039/c7sc00732a

**Published:** 2017-05-23

**Authors:** M. A. Rajora, L. Ding, M. Valic, W. Jiang, M. Overchuk, J. Chen, G. Zheng

**Affiliations:** a Princess Margaret Cancer Centre , University Health Network , 101 College Street , Toronto , Ontario M5G 1L7 , Canada . Email: gzheng@uhnresearch.ca; b Institute of Biomaterials and Biomedical Engineering , University of Toronto , 164 College Street , Toronto , Ontario M5S 3G9 , Canada; c Department of Medical Biophysics , University of Toronto , 101 College Street , Toronto , Ontario M5G 1L7 , Canada

## Abstract

The development of curative glioblastoma treatments and tumour-specific contrast agents that can overcome the blood–brain barrier (BBB) and infiltrative tumour morphology remains a challenge. Apolipoprotein E3 (apoE3) is a high density lipoprotein apolipoprotein that chaperones the transcytosis of nanoparticles across the BBB, and displays high-affinity binding with the low density lipoprotein receptor (LDLR), a cell-surface receptor overexpressed by glioblastoma cells. This LDLR overexpression and apoE3 binding capacity was exploited for the development of glioblastoma-targeted porphyrin-lipid apoE3 lipid nanoparticles (pyE-LNs) with intrinsic theranostic properties. Size-controlled discoidal and cholesteryl oleate (CO)-loaded spherical pyE-LNs were synthesized through the systematic variation of particle composition, which dictated nanoparticle size and morphology. Composition optimization yielded 30 nm pyE-LNs with stable loading of apoE3 and porphyrin-lipid that simultaneously conferred the nanoparticles with glioblastoma targeting and activatable near-infrared fluorescence imaging functionalities. A 4-fold higher uptake of pyE-LNs by LDLR-expressing U87 glioblastomas cells relative to minimally expressing ldlA7 cells was observed *in vitro*. This uptake was a result of receptor-mediated endocytosis, which could be inhibited through LDL competition and acetylation of particle apoE3 moieties. ApoE3-dependent delivery of pyE-LN to glioblastomas was also demonstrated in orthotopic U87-GFP tumour-bearing animals. Quantification of CO-loaded pyE-LN biodistribution demonstrated successful selective uptake of porphyrin by malignant tissue, with a 4 : 1 tumour : healthy tissue particle specificity. This allowed for the detection of strong, tumour-localized porphyrin fluorescence, which was diminished when apoE3-devoid py-LN particles were administered. Furthermore, this selective uptake yielded cell-specific potent PDT sensitization *in vitro*, resulting in an 83% reduction in glioblastoma cell viability. These results highlight the promising capacity of pyE-LNs to target porphyrin delivery to glioblastoma tumours for theranostic applications.

## Abstract 2

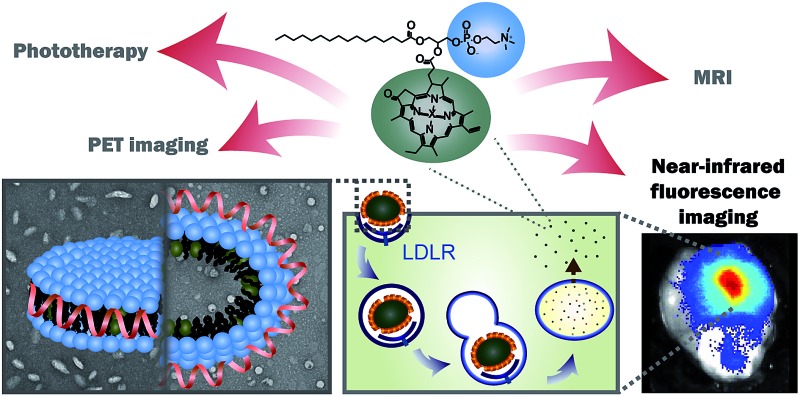
Size-controlled discoidal and cholesteryl oleated-loaded spherical, intrinsically multimodal porphyrin-lipid nanoparticles targeted glioblastoma *via* apoE3 and LDLR.

## Introduction

High grade gliomas, termed glioblastomas, remain a highly lethal disease with a mere 5% five-year survival rate.^1^ The aggressively invasive, heterogenous nature of these brain tumours prevent current treatment paradigms (surgical tumour resection and adjunctive radio or chemotherapy) from achieving successful remission, leading to inevitable recurrence.^1^ The development of accurate imaging modalities and curative glioblastoma interventions is challenged by the blood–brain barrier (BBB). Despite the presence of leaky vasculature within solid glioma tumours, the small 12 nm upper-limit size of these pores, in addition to the sustained presence of an intact BBB within the infiltrative tumour component, prevents the extravasation of contrast agents and drugs to all areas of the tumour.^2–4^ Furthermore, the diffuse infiltration of glioblastoma cells into healthy brain parenchyma not only prevents complete surgical resection of tumours,^5^ but also requires the use of imaging and therapeutic agents with high glioblastoma cell selectivity for accurate tumour delineation and prevention of off-target toxicity. Consequently, there exists an immediate need for therapeutic and imaging platforms that can cross the BBB and target glioma cells.

To this end, apolipoprotein E3 (apoE3) nanoparticles have gained attention as promising glioblastoma drug delivery vehicles. ApoE3 is one of three polymorphisms of the 34 kDa apoE glycoprotein; the most prominent lipoprotein produced within the central nervous system, where as a component of high-density lipoprotein (HDL), it regulates cholesterol and lipid homeostasis.^6,7^ Recently, apoE3 nanoparticles were shown to cross the BBB *via* transcytosis and exert neuroprotective effects in animal models of Alzheimer's disease.^8–12^ Furthermore, the transport of non-proteinaceous gold and polymeric nanoparticles is thought to be chaperoned by endogenous apoE adsorbed onto particle surfaces during their circulation in the blood stream.^13,14^ This mediation of nanoparticle active transport into the brain is thought to be in part due to the high-avidity binding of apoE3 to the low density lipoprotein receptor (LDLR) expressed in brain endothelium.^15,16^

The BBB permeability of apoE3 nanoparticles and this high affinity LDLR binding capacity makes this an interesting class of nanoparticles to explore as glioblastoma-targeted drug delivery vehicles. Several tumour types, including gliomas, require increased cholesterol metabolism to support lesion growth.^17,18^ This results in an upregulation of cell-surface LDLR, which facilitates cellular cholesterol uptake *via* receptor-mediated endocytosis of lipoproteins. Consequently, LDLR is overexpressed in human glioma cells compared to surrounding normal brain tissue.^19^ Recently, a limited number of studies exploited this LDLR overexpression for the delivery of apoE3-vitamin D3 nanoparticles and resveratrol, curcumin or DNA-loaded apoE3-HDL to glioblastoma cells.^20–23^ Though these studies demonstrated effective drug delivery *in vitro*, more extensive exploration of targeting specificity *in vitro* and *in vivo* is needed to further validate the utility of apoE3 nanoparticles for glioblastoma drug delivery.

**Fig. 1:**
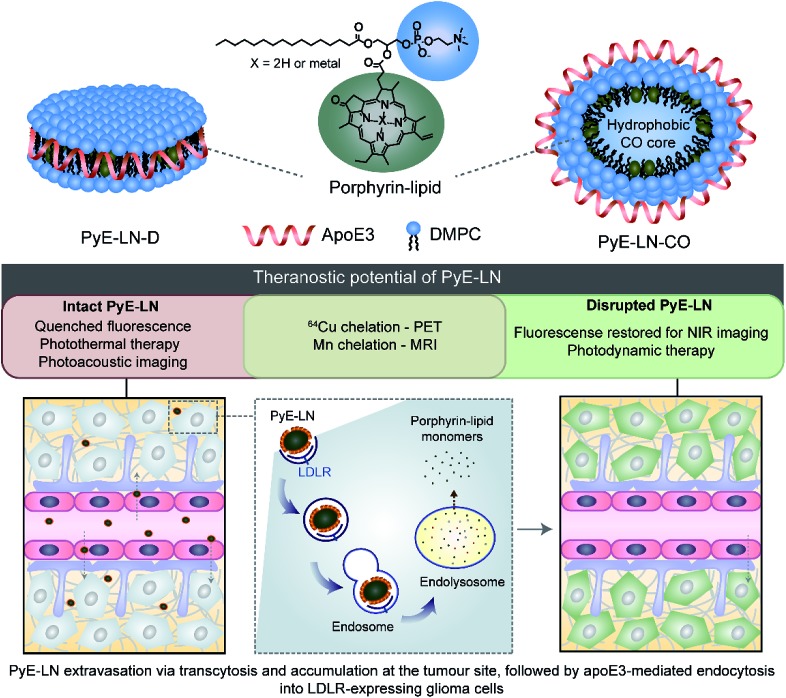
PyE-LN particles were formulated to comprise 1,2-dimyristoyl-*sn-glycero*-3-phosphocholine (DMPC) and porphyrin-lipid within the particle shell, assembled and size-constrained by apoE3 in either a discoidal or cholesteryl oleate (CO) core-loaded state, which represent two predominant populations of native HDL. It is anticipated that particles will permeate the BBB, accumulate at the tumour site, and undergo apoE3-directed LDLR-targeted uptake by glioma cells. Here, the multifunctional properties of porphyrin-lipid can be utilized for theranostic purposes. Intact particles display quenched fluorescence, giving rise to photoacoustic imaging and photothermal therapy capabilities. Following targeted endolysosomal uptake into glioma cells, particles are disrupted and monomeric porphyrin-lipid is released intracellularly. This dissociation restores porphyrin fluorescence to yield NIR imaging and photodynamic therapy capabilities.

In this study, we take advantage of the LDLR binding affinity of apoE3 and the overexpression of LDLR in malignant cells to address current needs in glioblastoma therapy and imaging *via* the development of porphyrin apoE3 lipid nanoparticles (pyE-LNs). Porphyrins are heterocyclic organic molecules that exhibit photophysical properties conducive to cancer fluorescence imaging and photodynamic therapy (PDT).^24^ Their integration into the shell of nanoparticles as lipid conjugates yields stable supramolecular structures with unique intrinsic multimodal (photoacoustic, near infrared fluorescence, positron emission tomography, magnetic resonance) imaging and phototherapy capabilities.^25–28^ By integrating porphyrin-lipid and apoE3 within a single nanostructure (Fig. 1), we expanded upon the currently limited repertoire of glioma-targeted apoE3 nanoparticles *via* the introduction of intrinsic all-in-one theranostic properties. Here, we present the development and characterization of these size-controlled pyE-LNs, and demonstrate their ability to specifically target glioblastoma cells in an apoE3-dependent manner *in vitro* and *in vivo*.

## Results and discussion

### Tailoring the size and composition of pyE-LN

Native HDL is comprised of a heterogeneous mixture of particle subclasses that range from dense discs to spherical particles core-loaded with cholesteryl esters and triglycerides, and which exhibit differing physiochemical and biological properties.^29–33^ To account for this variability, pyE-LNs were synthesized as discs (pyE-LN-D), comprising phospholipid, porphyrin-lipid and apoE3, or additionally loaded with cholesterol-oleate (pyE-LN-CO) to adopt a more spherical particle morphology (Fig. 1). Size and composition play important roles in the pharmacokinetic profiles of nanoparticles. Smaller subclasses of HDL exhibit higher serum stability and slower blood clearance relative to larger HDL populations.^34,35^ Furthermore, smaller nanoparticles permeate through the sub-40 nm extracellular matrix fibre spacing in glioblastoma tumours more effectively than their larger counterparts.^2,36–38^ As such, in order to promote pyE-LN stability and delivery while simultaneously capitalizing on the theranostic and translational potential of the shell-loaded porphyrin, pyE-LN compositions were tailored to minimize particle size and heterogeneity, and maximize porphyrin-lipid and CO loading.

**Fig. 2:**
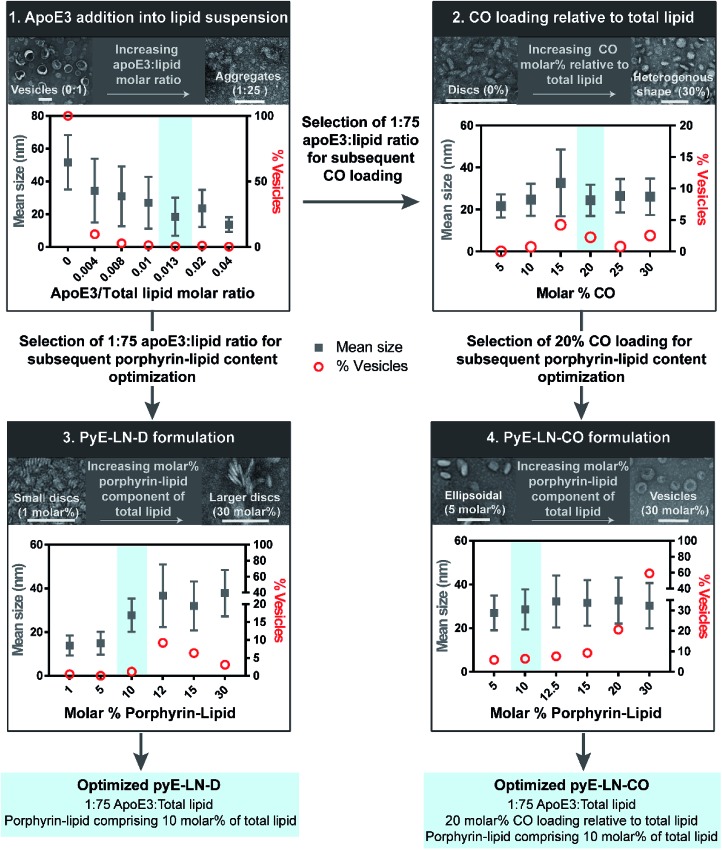
Optimization of pyE-LNs was performed *via* the systematic modification of particle composition. The molar ratio of DMPC and apoE3 was first varied to formulate discoidal particles which were then loaded with CO. Selection of optimal apoE3 : DMPC and CO/DMPC molar ratios was followed by the addition of porphyrin-lipid to the formulations, constituting up to 30 molar% of the total lipid content. Particles were imaged with TEM and analysed for morphology and size *via* ImageJ. Sizes are displayed as the average ± standard deviation of a minimum of 450 particle measurements from a minimum of three representative fields of view. Representative TEM images and histograms of all particle formulations are presented in Fig. S1–S4.† Scale bars = 100 nm.

Systematic optimization of the pyE-LNs, illustrated in Fig. 2, was conducted to achieve these design criteria. As the composition, and particularly the protein : lipid ratio, within HDL subclasses is associated with their size,^32,39,40^ the first step in this optimization process involved the variation of the apoE3 : total lipid molar ratio. ApoE3/1,2-dimyristoyl-*sn-glycero*-3-phosphocholine (DMPC) particles were synthesized by sonicating hydrated DMPC lipid films, yielding aqueous lipid vesicle suspensions to which apoE3 was added to consume and size-constrain the vesicles into proteinaceous discs. Increasing the apoE3 : lipid ratio decreased the vesicle population (Fig. 2 and S1†); an important consideration during particle formation as these vesicles, devoid of apoE3, lack targeting moieties and thus effectively increase particle impurity, while also increasing morphological heterogeneity and average particle size. To this end, increasing the apoE3 : lipid ratio also decreased particle size, a trend consistent with what is observed for native HDL.^32,39,40^ Beyond a 1 : 50 apoE3 : lipid molar ratio, protein aggregates were visible by TEM (Fig. S1†). As such, when taking lipid vesicle consumption, lack of protein aggregation, and the formation of small, morphologically-homogenous particles into account, a 1 : 75 apoE3 : lipid molar ratio was selected for further steps in the pyE-LN optimization process. Similar endpoints were also considered when subsequently selecting a 5 : 1 DMPC : CO molar ratio for the generation of pyE-LN-CO, whereby CO loading into the DMPC film resulted in a disc to ellipse/sphere shape change (Fig. 2 and S2†).

Lastly, porphyrin-lipid was added into DMPC or DMPC/CO lipid films to formulate optimized pyE-LN-D and pyE-LN-CO respectively using the total apoE3, lipid and CO compositions selected as described above in steps 1 and 2 of the optimization process (Fig. 2, S3 and S4†). Interestingly, increasing the porphyrin-lipid loading yielded differing trends for the two pyE-LN formulations: disc size increased with increasing porphyrin-lipid loading in pyE-LN-D, while particle size remained constant, but the vesicle population increased with higher loading of porphyrin-lipid into pyE-LN-CO. In both cases, a 90 molar% DMPC/10 molar% porphyrin-lipid composition was selected for optimal size-controlled particle formation. Overall, the results of this optimization process indicated that pyE-LN composition governed particle size and morphology, which could be tuned through the variation of lipid, apoE3 and CO molar ratios.

### Characterization of optimized pyE-LN

**Fig. 3:**
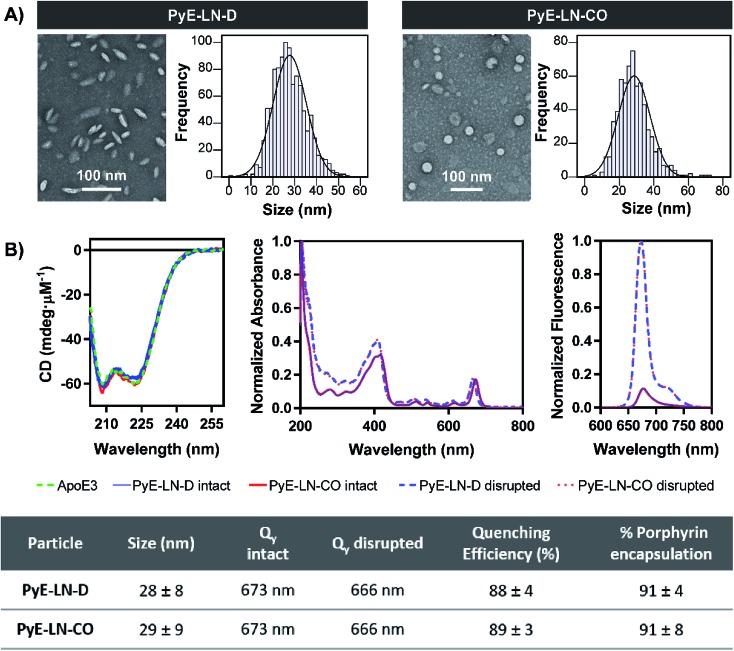
Characterization of the optimized pyE-LN-D and pyE-LN-CO. Particle size and morphology were assessed by TEM (A), while optical properties (B) were assessed using CD spectroscopy (presented as mdeg μM^–1^ of apoE3), spectrophotometry (intact particles measured in PBS, disrupted in methanol), and spectrofluorometry (410 nm excitation, disrupted particles measured in 1 v/v% Triton X-100, intact particles measured in PBS). Size is presented as an average ± standard deviation of a minimum of 450 ImageJ particle measurements from three sample replicates over three or more fields of view. Quenching efficiency and porphyrin encapsulation are presented as the average ± standard deviation of five samples. Absorbance and fluorescence profiles are normalized to the maximum absorbance and fluorescence intensity respectively in each spectra.

Following the identification of optimal apoE3, DMPC, porphyrin-lipid and CO compositions, tailored pyE-LNs were reproduced, and their physical chemical properties were characterized. As illustrated in Fig. 3A, a mean particle size of approximately 30 nm was achieved for optimized pyE-LN-D and pyE-LN-CO particles; a size potentially amenable to diffusion through the sub-40 nm spacing between glioblastoma tumour collagen fibrils,^37^ and consistent with sizes obtained for BBB-permeating apoE3/DMPC nanoparticles previously reported.^11,12^ Size distributions attained by TEM demonstrated the formation of monodisperse particles, wherein pyE-LN-D samples were composed of discs (Fig. 3 and S3†), while pyE-LN-CO samples were composed predominantly of spherical and more elliptical discoidal particles (Fig. 3 and S4†), a mixture observed consistently in previously reported TEM and cryo-TEM images of reconstituted HDL.^41,42^

Optical characterization of discoidal and CO-loaded pyE-LN demonstrated successful incorporation of apoE3 and porphyrin-lipid within the particles. Both nanoparticles, as illustrated in Fig. 3B, yielded circular dichroism (CD) spectra with a signature double peak between the 200 and 230 nm ultraviolet light range characteristic of the alpha-helical secondary structure of apoE in reconstituted HDL.^43^ CD values of –57.4 and –60.9 mdeg μM^–1^ protein were observed respectively for pyE-LN-D and pyE-LN-CO, similar to that of concentration-equivalent free apoE3 (–60.0 mdeg μM^–1^), demonstrating that apoE3 was present in the particle suspensions. Native gradient polyacrylamide gel electrophoresis of the pyE-LN samples demonstrated co-localization of protein and porphyrin fluorescence bands and stronger staining of particulate apoE3 bands *versus* those of free apoE3 (Fig. S5†). These observations illustrated that apoE3 within the particle suspensions was successfully co-assembled alongside porphyrin-lipid. Furthermore, a slight red-shift (7 nm) of the Q-band within the absorbance spectra of the intact particles was observed relative to the Q-band associated with disrupted particles (Fig. 3B). A similar red shift of 4 nm was noted in the fluorescence spectra of the intact particles relative to disrupted particles. Combined with an achievement of high porphyrin loading efficiency (∼90%) and porphyrin fluorescence quenching (∼90%) in intact particles, these observations indicated the presence of strong ground state coupling and the successful, stable assembly of porphyrin-lipid within both discoidal and CO-loaded pyE-LN.^44^ These assemblies remained stable under storage conditions (4 °C in PBS), whereby no significant difference (*n* = 3, *p* > 0.05) in quenching efficiency was observed for 30 days following particle synthesis (Fig. S6†). Particle size and morphology also remained stable over a period of 30 days (Fig. S6†), with no significant increase in size (*n* = 3, *p* > 0.05), indicating a lack of instability-induced particle aggregation. Thus, optimization of pyE-LN synthesis generated small (30 nm), monodisperse, stable pyE-LN-D and pyE-LN-CO particles loaded effectively with apoE3 and porphyrin-lipid.

In addition to remaining stable under storage conditions, discoidal and CO-loaded pyE-LNs maintained over 97% of their initial fluorescence quenching efficiencies over a 24 hour period at 37 °C in serum solutions composed of 10% FBS, and approximately 80% of their quenching efficiencies in 50% FBS solutions (Fig. S6†). This serum stability allows the activatable fluorescence of pyE-LN to be feasibly applied for theranostic purposes. As illustrated in Fig. 1, the fluorescence self-quenching of pyE-LN holds theranostic advantages: following the LDLR-targeted apoE3-mediated uptake of particles by glioblastoma cells, it is proposed that particles disassemble within endolysosomes into monomeric porphyrin-lipid with restored fluorescence, which among other applications, can be exploited to delineate tumours *via* near infrared (NIR) fluorescence imaging. The proposed utility of this target-specific activatable fluorescence was contingent on and conserved by the production of pyE-LNs stable under physiological conditions.

### Targeted *in vitro* glioblastoma cellular uptake of pyE-LN

**Fig. 4:**
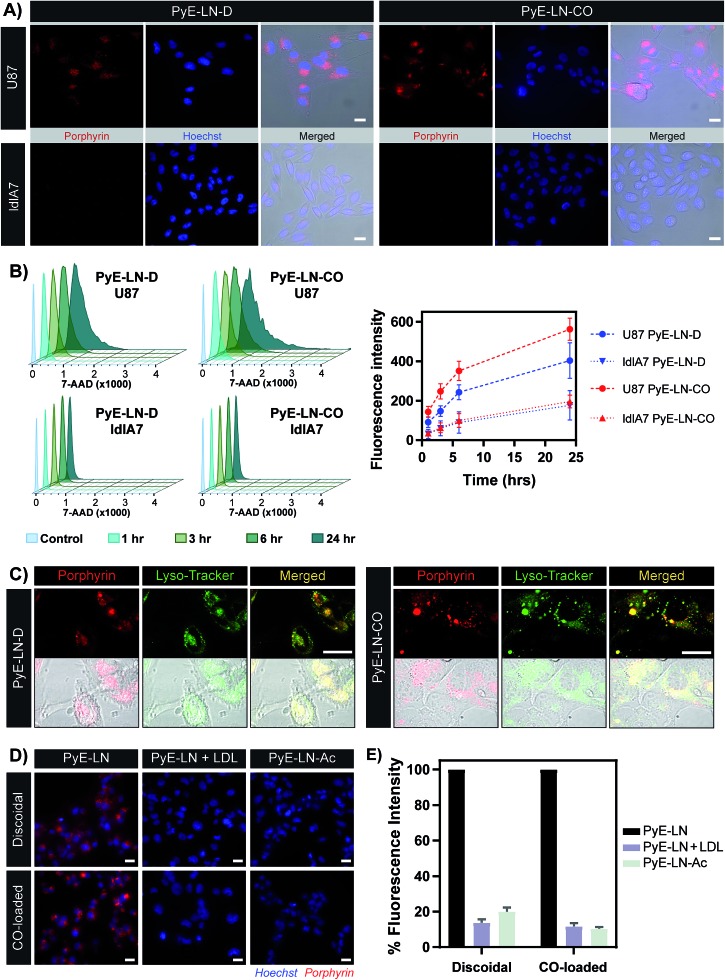
*In vitro* cell uptake studies of pyE-LN (5 μM porphyrin treatment concentration). (A) Comparison of pyE-LN uptake by U87 (high LDLR expression) and ldlA7 (low LDLR expression) cells with fluorescence microscopy following incubation with particles for 3 hours. (B) Representative flow cytometry histograms and associated quantification of time-dependent porphyrin uptake. At each time point, U87 cells displayed significantly higher porphyrin uptake than ldlA7 cells, while pyE-LN-CO demonstrated higher uptake than pyE-LN-D (*p* < 0.05, *n* = 4). (C) Confocal imaging of Lyso-Tracker-stained U87 cells following incubation with particles for 6 hours. (D and E) Treatment of U87 cells with pyE-LN, pyE-LN + 50× excess LDL or acetylated pyE-LN. Fluorescence microscope images (D) and flow cytometry quantification of cell uptake normalized to pyE-LN fluorescence intensity (E) are shown, with the latter demonstrating a significant decrease in absolute porphyrin fluorescence intensity (*p* < 0.005, *n* = 3) with LDL competition or particle acetylation for both pyE-LN-D and pyE-LN-CO (representative flow cytometry histograms are shown in Fig. S10†). Scale bars = 20 μm.

The proposed targeting ability of pyE-LNs was evaluated *in vitro* using human U87 glioblastoma and Chinese hamster ovary ldlA7 cells, which exhibited high and low levels of LDLR expression respectively (Fig. S7†). Activation of porphyrin fluorescence upon cellular delivery of the particles enabled the qualitative assessment and comparison of pyE-LN uptake between U87 and ldlA7 cells *via* fluorescence microscopy (Fig. 4A). Strong porphyrin fluorescence was observed in U87 cells treated with either discoidal or CO-loaded pyE-LN, while negligible fluorescence signal was detected in ldlA7 cells at equivalent exposure times, suggesting that pyE-LN uptake was facilitated by LDLR. This was further confirmed through flow cytometry quantitative measurements of porphyrin uptake in the two cell types exposed to either pyE-LN-D or pyE-LN-CO over a 24 hour period. Porphyrin fluorescence intensity increased qualitatively (Fig. S8†) and quantitatively in a time-dependent manner for discoidal and CO-loaded particles in both U87 and ldlA7 cells (Fig. 4B). However, particle uptake was 3–4-fold and 2–3 fold higher in U87 cells than ldlA7 cells for pyE-LN-CO and pyE-LN-D respectively (*n* = 4, *p* < 0.05). Interestingly, despite the two particles having similar average sizes, quenching efficiencies, serum stability, porphyrin loading, and qualitative time-dependent increase in cell uptake by U87 and ldlA7 cells (Fig. S8†), flow cytometry quantification of porphyrin uptake in U87 cells demonstrated a 1.5-fold higher (*n* = 4, *p* < 0.05) porphyrin fluorescence intensity following treatment with pyE-LN-CO *versus* pyE-LN-D at each time-point tested. This increased uptake of the more spherical variant of pyE-LN was consistent with previous findings of enhanced glioblastoma cell uptake of spherical apoE3-HDL particles compared to discoidal apoE3-HDL.^45^ The tertiary structure and microenvironment of apoE influences its LDLR binding avidity.^46,47^ The microenvironment of lysine residues and thereby apoE conformation is known to differ between discoidal and spherical apoE3 lipid nanoparticles.^48^ As such, although apoE3 within pyE-LN-D and pyE-LN-CO bears a similar secondary structure per CD spectroscopy, it is plausible that differences in the tertiary structure of apoE3 in the two pyE-LN variants leads to differential LDLR binding and subsequent uptake by the LDLR-expressing U87 cells. Nevertheless, the qualitative and quantitative observation of increased delivery of both pyE-LNs to U87 cells relative to ldlA7 cells indicates that the particles undergo cell-specific uptake.

Binding of a ligand to LDLR results in its receptor-mediated endocytosis followed by lysosomal fusion and degradation.^49,50^ Thus, in order to further substantiate whether the U87 cell-specific uptake of pyE-LNs was indeed a result of LDLR-mediated endocytosis, particles were incubated with U87 cells and stained with Lyso-Tracker. Confocal microscopy revealed a punctate pattern of porphyrin uptake that co-localized with the Lyso-Tracker signal (Fig. 4C), indicating that discoidal and CO-loaded pyE-LNs were sequestered into late endosomes or lysosomes, as would be expected following LDLR-mediated endocytosis. This potential delivery mechanism was further elucidated through the successful competitive inhibition of pyE-LN uptake in U87 cells by LDL. The strong fluorescence signal observed in U87 cells following a 3 hour treatment with either pyE-LN-D or pyE-LN-CO was eliminated when a 50-fold excess of LDL was added to the treatment media (Fig. 4D). Flow cytometry quantification revealed that LDL addition significantly reduced pyE-LN-D and pyE-LN-CO uptake in U87 cells by 86 ± 2% and 88 ± 2% respectively (*n* = 3, *p* < 0.005). PyE-LNs were thus shown to undergo LDLR-mediated uptake by glioblastoma cells.

Basic residues within the N-terminal domain of apoE3 are known to direct apoE3-LDLR binding, which is attenuated with the chemical modification of these residues.^51,52^ Consequently, acetylated pyE-LNs (PyE-LN-Ac) were synthesized and assessed *in vitro* to establish the role played by apoE3 in the glioma cell targeting abilities displayed by pyE-LN. Acetylation of protein moieties was conducted subsequent to particle formation in order to evade apoE3 modification-induced changes in particle self-assembly. Physical chemical properties of pyE-LNs, including particle size, apoE3 secondary structure, apoE3 and porphyrin-lipid incorporation, and quenching efficiencies, were conserved following acetylation (Fig. S5 and S9†). As was observed for LDL competition, acetylation of apoE3 moieties in pyE-LNs decreased the intracellular porphyrin signal observable by fluorescence microscopy following the treatment of U87 cells with pyE-LN-Ac (Fig. 4D). Acetylation significantly (*n* = 3, *p* < 0.005) decreased porphyrin uptake in U87 cells by 80 ± 2% and 90 ± 1% for discoidal and CO-loaded particles respectively, indicating that apoE3 mediated the *in vitro* LDLR-targeting abilities of pyE-LNs.

### *In vivo* glioblastoma tumour homing and theranostic utility

**Fig. 5:**
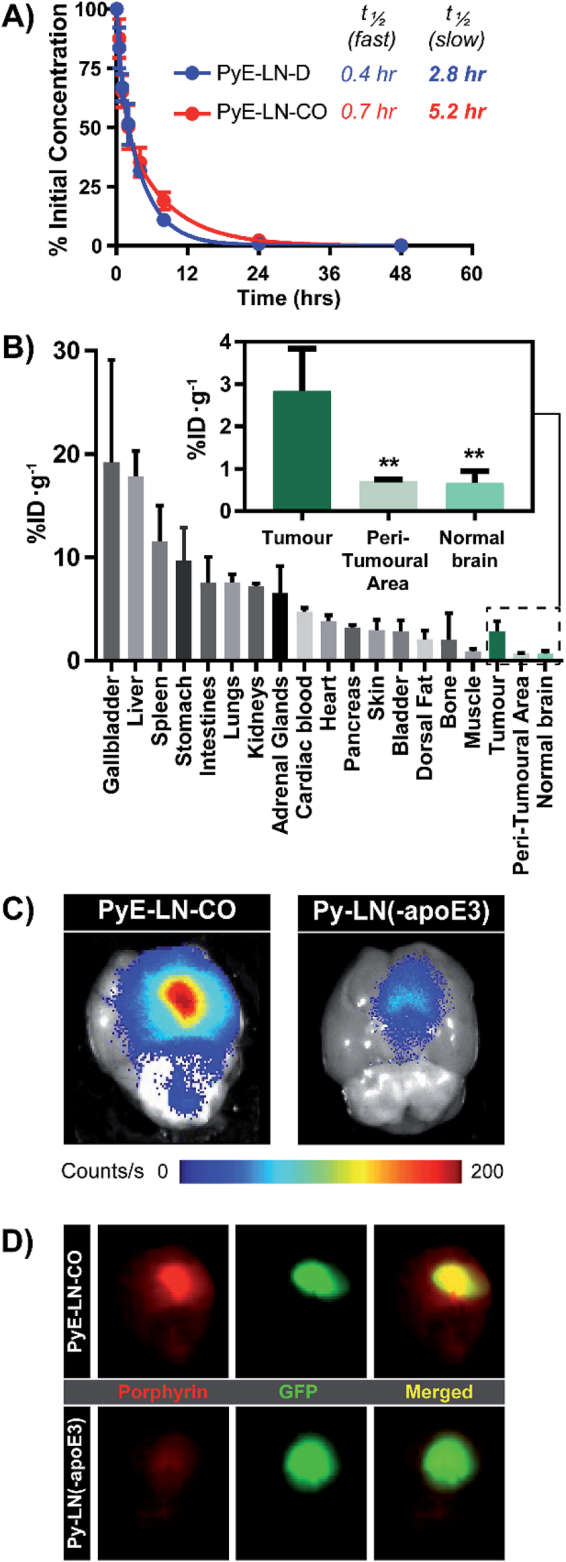
*In vivo* evaluation of pyE-LN. (A) Blood clearance profiled of pyE-LN formulations, and associated half-lives in healthy mice (*n* = 5). (B) Porphyrin biodistribution in U87-GFP tumour-bearing mice 6 hours following the administration of ^64^Cu-pyE-LN-CO (average ± standard deviation, *n* = 6). Significantly higher porphyrin accumulation (**p < 0.005, *n* = 6) was observed at the tumour site compared to surrounding tissue. Comparison of *ex vivo* fluorescence imaging of porphyrin signal intensity (C) and GFP signal co-localization (D) in brains harvested from U87-GFP tumour-bearing mice administered ^64^Cu-pyE-LN-CO or porphyrin dose equivalent py-LN(-apoE3).

The apoE3-mediated glioblastoma-targeting capacity of pyE-LN was subsequently characterized *in vivo* using U87-GFP orthotopic tumour-bearing mice. Due to its superior uptake by U87 cells *in vitro* and longer circulation half-life relative to discoidal particles (Fig. 5A), pyE-LN-CO was used for *in vivo* studies. By taking advantage of the intrinsic metal chelation abilities of porphyrin centres, pyE-LN-CO was radiolabelled with copper-64, yielding ^64^Cu-pyE-LN-CO, and allowing for the quantitative assessment of particle biodistribution *via* gamma counting. Importantly, the intrinsic ^64^Cu radiolabelling of porphyrin-lipid nanostructures does not necessitate the incorporation of added imaging agents, allowing the photophysical and biological properties of the parent particles to be conserved.^53^ Accordingly, ^64^Cu-pyE-LNs were synthesized with high radiolabelling efficiency (100 ± 1.6%) while maintaining the physical chemical properties of unlabelled pyE-LN-CO (Fig. S11†).

As illustrated in Fig. 5B, ^64^Cu-pyE-LN-CO accumulated to the greatest extent in the liver, gallbladder, spleen and gastrointestinal tract. This pattern of accumulation is consistent with the high expression of LDLR in the liver, stomach and intestinal tissue, the hepatobiliary clearance of porphyrins, sequestration of nanoparticles by the mononuclear phagocytic system, and with previously reported biodistribution of apoE3/lipid nanoparticles.^54–56^ ^64^Cu-pyE-LN-CO accumulated in tumour tissue at 3 ± 1% injected dose per gram of tissue. This accumulation was highly targeted to glioblastoma tissue, with a 4 : 1 tumour : peri-tumoral and tumour : contralateral normal brain tissue specificity. To the best of our knowledge, this is the first study to quantitatively establish the *in vivo* glioblastoma targeting specificity of synthetic apoE3 nanoparticles.

This tumour-homing ability was further evaluated *via* the activatable NIR fluorescence imaging capacity of pyE-LN-CO. *Ex vivo* fluorescence imaging of brains harvested from U87-GFP tumour-bearing mice 6 hours following the administration of ^64^Cu-pyE-LN-CO demonstrated strong porphyrin fluorescence at the tumour site that highly co-localized with U87 GFP signal (Fig. 5C and D). This tumour-specific imaging functionality was compared to that of DMPC/porphyrin-lipid nanoparticles synthesized without apoE3 (py-LN(-apoE3)). These particles displayed a larger size range due to the lack of apoE3 to mediate size control, but optical properties consistent with those of pyE-LN-CO (Fig. S11†). In comparison to pyE-LN-CO, py-LN(-apoE3) yielded weak fluorescence signal at the tumour site with no observable co-localization with U87 GFP fluorescence, indicating that pyE-LN uptake was apoE3-dependent (Fig. 5C and D). Collectively, these results validate the remarkable *in vivo* glioblastoma targeting capacity of pyE-LN.

**Fig. 6:**
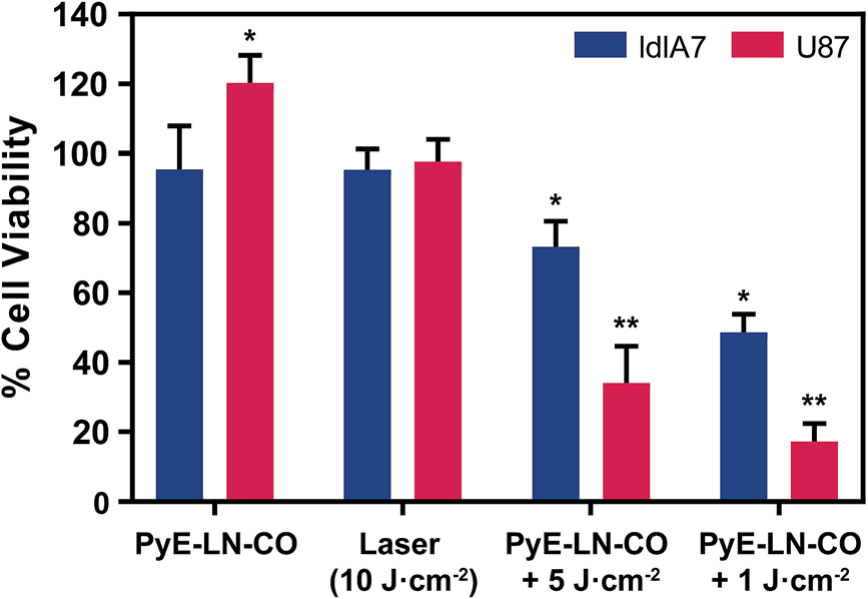
*In vitro* evaluation of pyE-LN PDT sensitization. Cell viability was normalized to untreated cells and is presented as the average of three replicates ± standard deviation. Cells were treated with py-LN-CO (3 μM), laser (671 nm) or a combination of laser and particle. Significant differences (*p < 0.01, *n* = 3) were observed between treated and untreated cells, wherein significantly higher toxicity (**p < 0.01, *n* = 3) was observed in U87 cells *versus* ldlA7 cells treated with particle and laser.

To further explore this targeting capacity and demonstrate the theranostic utility of pyE-LN, *in vitro* PDT was conducted in U87 and ldlA7 cells (Fig. 6). Laser or pyE-LN-CO administration alone yielded no cytotoxic effects against either cell line. In fact, pyE-LN-CO treatment of U87 cells enhanced cell viability by 20%. Cholesterol is an important substrate in sustaining cancer cell proliferation.^17^ As such, this augmentation of cell viability may be a result of increased cholesterol substrate delivery in the form of pyE-LN-CO, consistent with studies in which lipoprotein cholesterol treatment enhanced cancer cell proliferation.^57,58^ Nevertheless, co-treatment of U87 cells with pyE-LN-CO and low dose laser light yielded significant and highly potent cytotoxicity against U87 cells, wherein light doses of 1 and 5 J cm^–2^ yielded 67% and 83% cell death respectively. Such cytotoxic effects were markedly reduced by approximately 3-fold in ldlA7 cells co-treated with pyE-LN-CO and a 5 J cm^–2^ light dose, consistent with the higher expression of LDLR by U87 cells and the associated 3-fold reduced uptake of pyE-LN-CO by ldlA7 cells observed by flow cytometry (Fig. 4B) relative to U87 cells over a 24 hour treatment period. Thus, in addition to providing strong NIR fluorescence contrast *in vivo*, pyE-LN also demonstrated potent and specific phototherapeutic effects, making it a targeted and truly theranostic platform, which can be exploited in future studies to enhance *in vivo* glioblastoma PDT, and positron emission tomography and magnetic resonance imaging tumour contrast.

## Conclusions

The lack of curative interventions available for glioblastoma necessitates the development of platforms that can cross the BBB and specifically target malignant cells. PyE-LNs represent a novel class of theranostic vehicles that have the potential to address these needs. PyE-LN compositions could be tailored to generate size-controlled discoidal and spherical/elliptical CO-loaded particles with stable loading of porphyrin-lipid. These particles underwent LDLR-mediated endocytosis for the selective delivery of porphyrin to LDLR-overexpressing glioblastoma cells *in vitro*. Interestingly, CO-loaded pyE-LN displayed a longer blood clearance half-life and was more effectively uptaken than the discoidal variant by glioblastoma cells, suggesting that pyE-LN morphology influences pharmacokinetics and protein binding. The intrinsic multimodal properties of pyE-LNs were applied *in vivo* to illustrate the apoE3-dependent tumour-homing abilities of pyE-LN-CO, and *in vitro* to demonstrate potent PDT sensitizing capabilities. To the best of our knowledge, this is the first report of inherently multifunctional apoE3 nanoparticles with *in vivo* glioblastoma targeting capabilities. Furthermore, by establishing tumour specificity *in vivo*, this study validated the utility of apoE3 nanoparticles as glioma drug and contrast agent delivery platforms.

## Experimental

### PyE-LN synthesis

#### PyE-LN optimization

ApoE3 : total lipid, CO/total lipid and porphyrin-lipid/total lipid molar ratios were systematically modified in a 4-step optimization procedure for the synthesis of pyE-LNs (Fig. 2). Established procedures were followed during this optimization study.^12,28^ Briefly, 0.1–0.15 μmol lipid films were generated by combining appropriate quantities of DMPC (10 mg mL^–1^ in chloroform; Avanti Polar Lipids), CO (2.5 mg mL^–1^ in chloroform; Sigma Aldrich) and porphyrin-lipid (4–5 mg mL^–1^ in chloroform; pyropheophorbide/1-palmitoyl-2-hydroxy-*sn-glycero*-3-phosphocholine conjugate synthesized as described previously^25^) stock solutions into a glass vial, as summarized in Table 1. The chloroform was dried under nitrogen gas and then under vacuum to generate lipid films. The dried lipid films were re-hydrated with 1 mL phosphate buffered saline (PBS; 10 mM phosphate, pH 7.4) and subsequently bath-sonicated for 90 min. The resulting lipid solutions were allowed to passively cool to room temperature prior to the drop-wise addition of a 0.4 mg mL^–1^ apoE3 solution in PBS in a 75 : 1 total lipid : apoE3 molar ratio for steps 2, 3 and 4 of the optimization process and in a 1 : 0, 250 : 1, 125 : 1, 100 : 1, 75 : 1, 50 : 1 or 25 : 1 total lipid : apoE3 molar ratio for step 1 of the optimization procedure. The resulting solutions were slowly rotated overnight at 4 °C, after which the particle suspensions were centrifuged at 4 °C, 17 000*g* for 5 min to remove free porphyrin-lipid, DMPC or CO precipitates. The resulting supernatants were filtered through Corning® 0.2 μm syringe filters prior to characterization by TEM.

**Table 1:** Compositions of lipid films generated at each step within the pyE-LN optimization process

Step	DMPC (μmol)	Porphyrin-lipid (μmol)	CO (μmol)
1	0.15	—	—
2	0.15	—	0–0.03
3	0.1485	0.0015	—
	0.1425	0.0075	
	0.135	0.015	
	0.132	0.018	
	0.1275	0.0225	
	0.105	0.045	
4	0.095	0.005	0.02
	0.09	0.01	
	0.0875	0.0125	
	0.085	0.015	
	0.08	0.02	
	0.07	0.03	

#### Optimized pyE-LN-D and pyE-LN-CO synthesis

0.135 μmol of DMPC, 0.015 μmol of porphyrin-lipid, and in the case of pyE-LN-CO, 0.03 μmol of CO were combined as chloroform solutions in a glass vial. The chloroform was dried under nitrogen and subsequently under vacuum to form lipid films that were then hydrated with 1 mL of PBS. These hydrated films were bath sonicated for 90 min (48 °C for one hour, followed by Bioruptor sonication (low power, 30 second on/off cycles) for 30 min at 40 °C) to generate lipid suspensions. These suspensions were allowed to cool passively to room temperature prior to the drop-wise addition of 0.4 mg mL^–1^ apoE3 in a 75 : 1 total lipid : apoE3 molar ratio. The resulting particle suspensions were rotated overnight at 4 °C to allow for gentle mixing, after which free lipid and CO were precipitated *via* centrifugation at 4 °C, 17 000*g* for 5 min. The resulting supernatants were filtered through Corning® 0.2 μm syringe filters. PyE-LN solutions used for *in vitro* and *in vivo* studies were concentrated by 3 and 5–6-fold respectively using 10 000 NMWL regenerated cellulose Amicon® Ultra-15 centrifuge filter units prior to sterile filtration through Corning® 0.2 μm syringe filters. Particles were shielded from light and stored at 4 °C prior to use.

#### Synthesis of acetylated particles (pyE-LN-Ac)

Acetylation of pyE-LNs was conducted as previously described.^59^ 3 mL of pyE-LN particle solutions (7.39 μM apoE3) were stirred in glass scintillation vials cooled to 1 °C *via* an ice-water bath. A previously cooled 0.91 M 1-acetylimidazole (Aldrich) solution in PBS (1 mL; 100 mg 1-acetylimidazole) was added drop-wise to the pyE-LN solution. The contents of the vial were stirred for an additional minute prior to removal from the water bath. The reaction mixture was transferred to a 10 000 NMWL regenerated cellulose Amicon® Ultra-15 centrifuge filter unit, which was subsequently centrifuged at 4000*g*, 4 °C for 20 min. The resulting concentrate was diluted with 10 mL of PBS and centrifuged again. Washes were repeated until no 200–300 nm UV-Vis optical signatures of free imidazole were observed in the wash filtrate (this required 5–7 total washes). The final concentrate was then diluted up to a 1 mL volume with PBS, centrifuged in an Eppendorf tube at 17 000*g*, 4 °C for 5 min prior to sterile filtration through a Corning® 0.2 μm syringe filters. Particles were shielded from light and stored at 4 °C prior to use.

#### Synthesis of py-LN(-apoE3)

Py-LN(-apoE3) vesicles devoid of apoE3 were generated as previously described.^25^ Chloroform solutions of porphyrin-lipid, DMPC, cholesterol and distearoyl-*sn-glycero*-3-phosphoethanolamine-*N*-methoxy(polyetheneglycol) (PEG2000-DSPE) were combined in a 10 : 45 : 40 : 5 molar ratio in a glass vial. The chloroform was dried under nitrogen and vacuum to form lipid films, which were then hydrated with PBS. The hydrated films were subjected to 9 freeze/thaw cycles prior to extrusion *via* a LIPEX Thermobarrel Extruder through a polycarbonate membrane (100 nm pore size) 10 times at 65 °C to yield py-LN(-apoE3). The particles were sterile filtered through a Corning® 0.2 μm syringe filters, shielded from light and stored at 4 °C prior to use.

#### ^64^Cu radiolabelling of pyE-LN-CO (^64^Cu-pyE-LN-CO)^28^

Positron emitting ^64^CuCl_2_ was supplied in acidic aqueous buffer by Washington University Medical School (St. Louis, NO, USA). The ^64^CuCl_2_ was diluted to an activity concentration of 2.5 mCi mL^–1^ with freshly prepared, ion exchanged (demetallized) 0.1 M ammonium acetate (Aldrich) buffer, pH 5.5. The diluted ^64^CuCl_2_ was added to 1 μmol of a 6-fold concentrated solution of pyE-LN-CO (1 mL) to yield a final concentration of 2.5 mCi μmol^–1^ of porphyrin. This radiolabelling mixture was incubated in a 37 °C water bath for 60 min. Upon completion of labelling, each radiolabelled mixture was purified using 30 000 NMWL regenerated cellulose Amicon® centrifugal filter units to remove ammonium acetate buffer and non-chelated ^64^CuCl_2_. Two washes of the concentrate containing the ^64^Cu-labelled pyE-LN-CO were conducted with sterile PBS and the filtrate was recovered to characterize radiolabelling efficiency *via* gamma-counting. The resulting counts were used to calculated a radiolabelling efficiency of 100 ± 1.6% as follows:
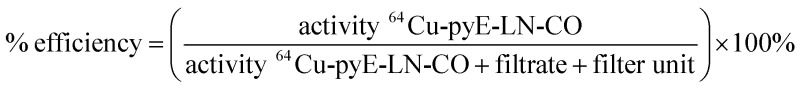


Lastly, the purified ^64^Cu-pyE-LN-CO nanoparticles were diluted in sterile PBS to yield a final activity concentration of approximately 1.0 mCi mL^–1^.

### PyE-LN characterization

#### TEM

TEM was conducted to assess particle size and morphology at direct magnifications of 80 000–150 000× using a FEI Techai 20 electron microscope (Nanoscale Biomedical Imaging Facility, Peter Gilgan Centre for Research and Learning, Toronto) equipped with a digital camera. Samples were adsorbed onto charged carbon grids (Electron Microscopy Sciences) and imaged following 2% uranyl acetate negative staining. Average particle sizes were obtained from the measurement of a minimum of 450 particles over three representative fields of view using ImageJ. The percentage of the particles with a vesicular morphology was determined by counting the total number of vesicles and particles over three representative fields of view for each particle formulations. Dynamic light scattering (Zetasizer, Malvern Instruments) was used as a supplementary technique to compare size distributions of acetylated and radiolabelled particles against parent pyE-LNs.

#### Optical characterization

Optical characterization of solutions containing either intact or disrupted particles was conducted using CD spectrometry (Jasco J18; 5 accumulations, measurements conducted in PBS and normalized to the theoretical apoE3 concentration), spectrofluorometry (Horiba Fluoromax-4; 410 nm excitation, 600–750 nm emission, 5 nm slit width), and UV-Vis spectrometry (Varian 50 Bio). All intact particle measurements were conducted in PBS. Particles were disrupted in 0.5–1 v/v% Triton-X 100 or methanol for fluorescence and UV-Vis measurements respectively. The porphyrin concentration and percent encapsulation in each sample were determined using the Beer–Lambert Law (porphyrin *ε* 45 000 M^–1^ cm^–1^ at 667 nm in methanol). Fluorescence quenching efficiency was calculated over a 600–750 nm emission range as follows:
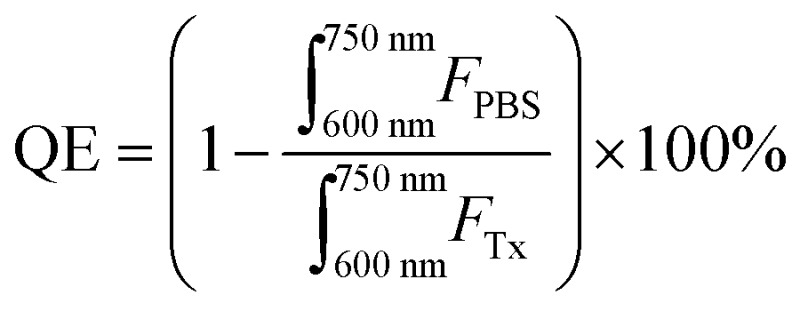
where, *F*_Tx_ and *F*_PBS_ represent fluorescence intensities of concentration-equivalent particle solutions in 0.5–1 v/v% Triton X-100 (disrupted particles) and PBS (intact particles) respectively.

#### Native gel electrophoresis

Native gel electrophoresis was conducted as previously described^60^ to ensure apoE3 in the particle suspensions was co-assembled with porphyrin-lipid. A Mini-Protean® precast 4–20% polyacrylamide gel (Bio-Rad) was preconditioned for 20 min at 125 V in a Tris (0.089 M), boric acid (0.089 M), EDTA (0.002 M) electrophoresis buffer (Bio Basic Inc.). Samples were diluted 1.25× in loading buffer (40% sucrose, 0.05% bromophenol blue aqueous solution) prior to loading into wells. Electrophoresis was conducted at 4 °C using the following voltages: 20 V for 15 min, 70 V for 20 min, 125 V for 17.2 h. The gel was removed from the chamber and porphyrin fluorescence was detected using a Cri Maestro-2 whole animal fluorescence imaging system (616–661 nm excitation, 675 nm-longpass emission, 750 ms exposure). The gel was subsequently stained to visualized protein bands using a Pierce® Silver Stain Kit (Thermo Scientific), and imaged in white light. Images were co-registered to assess porphyrin and apoE3 band co-localization.

#### Serum stability

Serum stability of pyE-LNs was evaluated *via* quenching efficiency measurements. Particle solutions (55 μM porphyrin concentration) were mixed with appropriate quantities of FBS or PBS to generate 0, 10 and 50 v/v% FBS solutions. These particle/FBS solutions were heated at 37 °C in a MultiTherm shaker (Benchmark Scientific). Aliquots were removed at 0, 1, 2, 4, 8 and 24 h for quenching efficiency measurements as described above.

### PyE-LN cell uptake and *in vitro* PDT studies

#### Cell culture

Human U87-MG and U87-MG-GFP glioblastoma cells were gifts from Dr Brian Wilson (Princess Margaret Cancer Centre, Toronto), human HepG2 hepatocellular carcinoma cells were purchased from ATCC and Chinese hamster ovary ldlA7 cells were acquired as a gift from Dr Monty Kreiger (Massachusetts Institute of Technology). U87 cells were cultured in Minimum Essential Medium (Gibco) supplemented with 10 v/v% FBS (WISENT). U87-GFP cells were cultured in Minimum Essential Medium supplemented with FBS (10%) and penicillin–streptomycin (1%). ldlA7 cells were cultured in Hams F-12 medium (with l-glutamine; Gibco) supplemented with penicillin–streptomycin (1 v/v%), FBS (5 v/v%) and l-glutamine (2 mM). HepG2 cells were cultured in Dulbecco's Modified Eagle Medium (Sigma Aldrich) supplemented with FBS (10 v/v%). All cells were cultured at 37 °C in a 5% CO_2_ atmosphere.

#### Western blot

Anti-LDLR antibody (ab52818) and HRP-conjugated anti β-actin antibody were purchased from Abcam (Cambridge, MA, USA). Both antibodies were diluted 1 : 5000 in 5% BSA/TBST. Five million U87, ldlA7 and HepG2 cells were collected and lysed in lysis buffer, from which protein samples were obtained and loaded into a 10% SDS-PAGE gels, and then transferred to a PVDF membrane (Bio-Rad). The membranes were subsequently blocked with 5% fat-free milk in TBST overnight. Membranes were then incubated with primary antibodies overnight at 4 °C, followed by incubation with 1 : 2000 diluted HRP-conjugated anti-rabbit antibody (Cell Signaling Technology) for 1 hour at room temperature. Detection of protein bands was performed by a Konica SRX-101A Medical Film Processor. The blots were stripped and re-probed with HRP-conjugated anti β-actin antibody as a loading control.

#### Fluorescence microscope imaging

U87 and ldlA7 cells were seeded into 8-well chambered coverglass systems (Lab-Tek) at respective cell-seeding densities of 2 × 10^4^ and 1.5 × 10^4^ cells per well. After a 24 hour incubation, medium was replaced and supplemented with 5 μM (by porphyrin concentration) pyE-LN-D or pyE-LN-CO. For inhibition studies, the following treatments were also delivered in separate wells: (1) pyE-LNs (5 μM porphyrin concentration) and LDL (isolated from human plasma, Sigma Aldrich; 50× by mass of protein relative to pyE-LNs); and (2) pyE-LN-Ac (5 μM porphyrin concentration). Cells were incubated for an additional 3 hours prior to the addition of 0.5 μL Hoechst 33258 nuclear stain (1 mg mL^–1^; Sigma) to the treatment medium. For time-dependent imaging studies, cells were incubated in treatment media for 1, 3, 6, or 24 hours. After 10 min of incubation with the Hoechst stain, medium was removed and wells were washed with PBS, medium and replenished with fresh medium prior to imaging at 60× magnification with an Olympus IX73 fluorescence microscope. Porphyrin signal was detected using a Cy5 filter cube (628/40 nm excitation, 692/40 nm emission), while Hoechst nuclear signal was detected using a DAPI filter cube (387/11 nm excitation, 447/60 nm emission). Light exposure times were kept consistent when imaging ldlA7 and U87 cells. Images were processed using ImageJ.

#### Quantitative analysis of pyE-LN uptake by flow cytometry

U87 and ldlA7 cells were seeded into 6-well plates at a 1 million cells per well density. Cells were incubated for 24 h prior to replenishing the wells with 0.5 mL of fresh media. Cells were subsequently treated with pyE-LNs at a 5 μM porphyrin concentration in the following groups: (1) 1 h incubation with pyE-LN-D or pyE-LN-CO treatment, (2) 3 h incubation with pyE-LN-D or pyE-LN-CO, (3) 3 h incubation with pyE-LN-D or pyE-LN-CO + 50× LDL, (4) 3 h incubation with pyE-LN-D-Ac or pyE-LN-CO-Ac, (5) 6 h incubation with pyE-LN-D or pyE-LN-CO, (6) 24 h incubation with pyE-LN-D or pyE-LN-CO, and (7) no treatment control. Cells were then trypsinized, centrifuged and washed 3 times with PBS prior to adding 500 μL FACS buffer (0.5 mM EDTA and 5 mg L^–1^ DNase in PBS). The resulting cell suspensions were filtered into 5 mL polystyrene test tubes equipped with cell strainer snap caps. Cells were then separated with a BD LSR II flow cytometer, and porphyrin fluorescence was detected using the 7 AAD channel (excitation 635 nm-longpass, emission 660/20 nm) for 10 000 events. Median fluorescence intensity was measured and corrected for background signal from control cells, and histograms were generated using FlowJo software.

#### Confocal imaging

U87 cells were seeded into 8-well chambered coverglass systems and treated with pyE-LNs for 6 h as described above for fluorescence microscope imaging. After 6 h of incubation, treatment medium was replaced by fresh medium, and the cells were incubated for an additional 18 h. Cells were then washed with PBS twice and wells were replenished with phenol red-free Earle's Minimum Essential Medium (EMEM) with l-glutamine (Quality Biological Inc.) supplemented with 10% FBS. LysoTracker Green DND-26 (ThermoFisher) was added to the wells at a final concentration of 0.5 μM. Following 3 min of incubation, cells were washed twice with PBS and wells were replenished with the 10% FBS phenol red-free medium. Images were acquired using an Olympus FV1000 laser confocal scanning microscope. Porphyrin signal was detected using a 653 nm excitation and 668–768 nm-emission, while LysoTracker signal was detected using a 488 nm excitation and 500–596 nm emission. Images were processed using ImageJ.

#### *In vitro* PDT

*In vitro* PDT experiments were conducted using pyE-LN-CO. U87 and ldlA7 cells were seeded at a density of 7000 cells per well into black 96-well plates. The treatment and control groups were as follows: (1) no laser and no particle control, (2) pyE-LN-CO treatment alone, (3) laser treatment (10 J cm^–2^) alone, (4) pyE-LN-CO + 1 J cm^–2^ laser treatment, and (5) pyE-LN-CO + 5 J cm^–2^ laser treatment. After 24 hours, medium was replaced with treatment medium consisting of pyE-LN-CO at a concentration of 3 μM in Minimum Essential Medium (Gibco) or Ham's F12 medium (with l-glutamine; Gibco) for the treatment of U87 and ldlA7 cells respectively. Wells containing cells receiving no treatment or laser treatment alone were replenished with the above medium devoid of pyE-LN-CO particles. After a 24 h incubation period, cells were washed with PBS and wells were replenished with fresh medium. PDT was administered using a continuous wavelength (671 nm) free-space laser (LaserGlow Technologies), with a 25 mW power output, and 0.7 cm diameter spot size. Light doses of 10, 5 and 1 J cm^–2^ were administered through laser irradiation for 200, 100 and 20 s respectively. Following PDT administration, cells were incubated for an additional 24 h, after which medium was replaced with that containing 0.5 mg mL^–1^ 3-(4,5-dimethylthiazol-2-yl)-2,5-diphenyltetrazolium bromide (MTT; Invitrogen). Cells were incubated for 2 h, medium was removed, and well contents were digested with 100 μL dimethyl sulfoxide. Plates were shaken prior to obtaining absorbance measurements at 570 nm using a CLARIOstar microplate reader (BMG LABTECH). Percent cell viability for each experiment was determined by normalizing the averaged blank-corrected absorbance values of 4 replicate treatment wells against blank-corrected absorbance values of wells administered medium alone. Each experiment was replicated in triplicate.

### *In vivo* pharmacokinetics and biodistribution of pyE-LNs

All in *in vivo* studies were approved and conducted in compliance with the University Health Network Animal Resource Centre guidelines.

#### Blood clearance of pyE-LNs

Established procedures were followed to evaluate the blood clearance of discoidal and CO-loaded pyE-LNs.^28^ Healthy female BALB/c mice were injected with pyE-LN-D or pyE-LN-CO at a porphyrin dose of 5 mg kg^–1^ *via* the tail vein (*n* = 5 per group). Blood was collected from the femoral vein with heparin-coated capillary tubes prior to and 5 min, 30 min, 1 h, 2 h, 4 h, 8 h, 12 h, 24 h and 48 h after particle injection. Plasma was collected following centrifugation of the blood samples. Samples were diluted in dimethyl sulfoxide 150× to disrupt any intact particles, and subsequently measured for porphyrin fluorescence using spectrofluorometry (Horiba Fluoromax-4; 650 nm excitation, 660–760 nm emission, 5 nm slit width). Fluorescence intensities over the emission range were summed, corrected for the summed intensity associated with blood samples collected prior to pyE-LN administration, and normalized to the 5 minute sample summed fluorescence intensity. Blood clearance was modeled using a two-compartment model in GraphPad Prism® to calculate slow and fast circulation half-lives.

#### Orthotopic U87-GFP glioma model

A previously established model was applied to characterize the biodistribution of pyE-LN-CO.^28^ Female athymic nude mice were anaesthetized under surgical plane, and placed within a stereotactic frame. A 1 mm diameter burr hole was created in the left cerebral hemisphere. U87-GFP cells (5 × 10^4^) were injected through the exposed dura into the underlying brain parenchyma. Upon wound closure, animals were administered 0.1 mg kg^–1^ buprenorphine analgesic and were monitored while recovering from anaesthesia. Tumour growth was monitored weekly by T_2_-weighted MRI. Animals were used for biodistribution studies when the longest axis of the tumour reached a length of 2–4 mm. Mice were placed on an autofluorescence-reduced diet 1 day prior to administering pyE-LN.

#### Quantitative biodistribution of ^64^Cu-pyE-LN-CO

Five mice bearing U87-GFP tumours were each administered 300 μL of freshly prepared radiolabelled particles through the tail vein with a corresponding dose of 0.3 mCi per animal and 6.75 mg kg^–1^ porphyrin. Six hours post administration, each mouse was euthanized using cardiac puncture. A complete tissue biodistribution was performed inclusive of all major organs, tumour, peri-tumoural area and normal brain tissue from the contralateral hemisphere. Each collected tissue and blood was weighed and measured for ^64^Cu gamma-activity using an automatic gamma counter (Perkin Elmer, Waltham, MA, USA) with a linear detection limit for ^64^Cu of 1.88 nCi (60 second integration time). Activity measurements were decay-corrected to the time of injection and expressed as a percentage of the injected dose per gram of tissue.

#### *Ex vivo* fluorescence imaging of pyE-LN tumour uptake

Prior to excising tumour tissue for gamma-counting, brains harvested from mice administered ^64^Cu-pyE-LN-CO were imaged *ex vivo* using the Maestro-2 whole animal fluorescence imaging system. Porphyrin fluorescence was detecting with a red filter (616–661 nm excitation, 675 nm-longpass emission, 150 ms exposure), while GFP signal was detected using a blue filter (435–480 nm excitation, 515–545 nm emission, 20 ms exposure). Images were processed using the Maestro software and ImageJ. A second subset of U87-GFP tumour-bearing animals were administered py-LN(-apoE3) at 6.75 mg kg^–1^ porphyrin. Six hours after particle administration, animals were euthanized, after which brains were harvested and subjected to Maestro imaging as described above.

### Statistics

Independent samples *t*-tests were used to compare the means of two groups (equal variances not assumed) with a *p* < 0.05 denoting statistical significance.

## References

[cit1] Ostrom Q. T., Gittleman H., Liao P., Rouse C., Chen Y., Dowling J., Wolinsky Y., Kruchko C., Barnholtz-Sloan J. (2014). Neuro-Oncology.

[cit2] Sarin H., Kanevsky A. S., Wu H., Brimacombe K. R., Fung S. H., Sousa A. A., Auh S., Wilson C. M., Sharma K., Aronova M. A., Leapman R. D., Griffiths G. L., Hall M. D. (2008). J. Transl. Med..

[cit3] Sarin H., Kanevsky A. S., Wu H., Sousa A. A., Wilson C. M., Aronova M. A., Griffiths G. L., Leapman R. D., Vo H. Q. (2009). J. Transl. Med..

[cit4] Brat D. J., Van Meir E. G. (2004). Lab. Invest..

[cit5] Giese A., Bjerkvig R., Berens M. E., Westphal M. (2003). J. Clin. Oncol..

[cit6] Bu G. (2009). Nat. Rev. Neurosci..

[cit7] Holtzman D. M., Herz J., Bu G. (2012). Cold Spring Harbor Perspect. Med..

[cit8] Michaelis K., Hoffmann M. M., Dreis S., Herbert E., Alyautdin R. N., Michaelis M., Kreuter J., Langer K. (2006). J. Pharmacol. Exp. Ther..

[cit9] Balducci C., Mancini S., Minniti S., La Vitola P., Zotti M., Sancini G., Mauri M., Cagnotto A., Colombo L., Fiordaliso F., Grigoli E., Salmona M., Snellman A., Haaparanta-Solin M., Forloni G., Masserini M., Re F. (2014). J. Neurosci..

[cit10] Zensi A., Begley D., Pontikis C., Legros C., Mihoreanu L., Wagner S., Buchel C., von Briesen H., Kreuter J. (2009). J. Controlled Release.

[cit11] Song Q., Song H., Xu J., Huang J., Hu M., Gu X., Chen J., Zheng G., Chen H., Gao X. (2016). Mol. Pharm..

[cit12] Song Q., Huang M., Yao L., Wang X., Gu X., Chen J., Chen J., Huang J., Hu Q., Kang T., Rong Z., Qi H., Zheng G., Chen H., Gao X. (2014). ACS Nano.

[cit13] Schaffler M., Sousa F., Wenk A., Sitia L., Hirn S., Schleh C., Haberl N., Violatto M., Canovi M., Andreozzi P., Salmona M., Bigini P., Kreyling W. G., Krol S. (2014). Biomaterials.

[cit14] Kreuter J. (2013). J. Microencapsulation.

[cit15] Sarkar G., Curran G. L., Mahlum E., Decklever T., Wengenack T. M., Blahnik A., Hoesley B., Lowe V. J., Poduslo J. F., Jenkins R. B. (2011). PLoS One.

[cit16] Lucarelli M., Borrelli V., Fiori A., Cucina A., Granata F., Potenza R. L., Scarpa S., Cavallaro A., Strom R. (2002). FEBS Lett..

[cit17] Cruz P. M., Mo H., McConathy W. J., Sabnis N., Lacko A. G. (2013). Front. Pharmacol..

[cit18] Villa G. R., Hulce J. J., Zanca C., Bi J., Ikegami S., Cahill G. L., Gu Y., Lum K. M., Masui K., Yang H., Rong X., Hong C., Turner K. M., Liu F., Hon G. C., Jenkins D., Martini M., Armando A. M., Quehenberger O., Cloughesy T. F., Furnari F. B., Cavenee W. K., Tontonoz P., Gahman T. C., Shiau A. K., Cravatt B. F., Mischel P. S. (2016). Cancer Cell.

[cit19] Rudling M. J., Angelin B., Peterson C. O., Collins V. P. (1990). Cancer Res..

[cit20] Maleklou N., Allameh A., Kazemi B. (2016). J. Drug Targeting.

[cit21] Kim S. H., Adhikari B. B., Cruz S., Schramm M. P., Vinson J. A., Narayanaswami V. (2015). PLoS One.

[cit22] Mousazadeh M., Palizban A., Salehi R., Salehi M. (2007). J. Drug Targeting.

[cit23] Ghosh M., Ryan R. O. (2014). Nanomedicine.

[cit24] Josefsen L. B., Boyle R. W. (2012). Theranostics.

[cit25] Lovell J. F., Jin C. S., Huynh E., Jin H., Kim C., Rubinstein J. L., Chan W. C. W., Cao W., Wang L. V., Zheng G. (2011). Nat. Mater..

[cit26] Liu T. W., MacDonald T. D., Jin C. S., Gold J. M., Bristow R. G., Wilson B. C., Zheng G. (2013). ACS Nano.

[cit27] MacDonald T. D., Liu T. W., Zheng G. (2014). Angew. Chem., Int. Ed..

[cit28] Cui L., Lin Q., Jin C. S., Jiang W., Huang H., Ding L., Muhanna N., Irish J. C., Wang F., Chen J., Zheng G. (2015). ACS Nano.

[cit29] Rothblat G. H., Phillips M. C. (2010). Curr. Opin. Lipidol..

[cit30] Davidson W. S., Sparks D. L., Lund-Katz S., Phillips M. C. (1994). J. Biol. Chem..

[cit31] Rosenson R. S., Brewer Jr H. B., Chapman M. J., Fazio S., Hussain M. M., Kontush A., Krauss R. M., Otvos J. D., Remaley A. T., Schaefer E. J. (2011). Clin. Chem..

[cit32] Skipski V. P., Barclay M., Barclay R. K., Fetzer V. A., Good J. J., Archibald F. M. (1967). Biochem. J..

[cit33] Asztalos B. F., de la Llera-Moya M., Dallal G. E., Horvath K. V., Schaefer E. J., Rothblat G. H. (2005). J. Lipid Res..

[cit34] Gao X., Yuan S., Jayaraman S., Gursky O. (2009). J. Mol. Biol..

[cit35] Lamarche B., Uffelman K. D., Steiner G., Barrett P. H., Lewis G. F. (1998). J. Lipid Res..

[cit36] Mo J., He L., Ma B., Chen T. (2016). ACS Appl. Mater. Interfaces.

[cit37] Pluen A., Boucher Y., Ramanujan S., McKee T. D., Gohongi T., di Tomaso E., Brown E. B., Izumi Y., Campbell R. B., Berk D. A., Jain R. K. (2001). Proc. Natl. Acad. Sci. U. S. A..

[cit38] Kafa H., Wang J. T., Rubio N., Klippstein R., Costa P. M., Hassan H. A., Sosabowski J. K., Bansal S. S., Preston J. E., Abbott N. J., Al-Jamal K. T. (2016). J. Controlled Release.

[cit39] Wilson H. M., Griffin B. A., Watt C., Skinner E. R. (1992). Biochem. J..

[cit40] Gidez L. I., Miller G. J., Burstein M., Slagle S., Eder H. A. (1982). J. Lipid Res..

[cit41] Tansey J. T., Thuren T. Y., Jerome W. G., Hantgan R. R., Grant K., Waite M. (1997). Biochemistry.

[cit42] Zhang L., Song J., Newhouse Y., Zhang S., Weisgraber K. H., Ren G. (2010). J. Lipid Res..

[cit43] Narayanaswami V., Maiorano J. N., Dhanasekaran P., Ryan R. O., Phillips M. C., Lund-Katz S., Davidson W. S. (2004). J. Biol. Chem..

[cit44] Luby B. M., Charron D. M., MacLaughlin C. M., Zheng G. (2016). Adv. Drug Delivery Rev..

[cit45] Huang J.-L., Gan J., Song Q.-X., Gu X., Song H.-H., Wang X.-L., Jiang D., Kang T., Feng X.-Y., Jiang X.-G., Chen H.-Z., Gao X.-L. (2016). Die Pharmazie.

[cit46] Lund-Katz S., Wehrli S., Zaiou M., Newhouse Y., Weisgraber K. H., Phillips M. C. (2001). J. Lipid Res..

[cit47] Gupta V., Narayanaswami V., Budamagunta M. S., Yamamato T., Voss J. C., Ryan R. O. (2006). J. Biol. Chem..

[cit48] Lund-Katz S., Weisgraber K. H., Mahley R. W., Phillips M. C. (1993). J. Biol. Chem..

[cit49] Anderson R. G., Brown M. S., Goldstein J. L. (1977). Cell.

[cit50] Goldstein J. L., Anderson R. G., Brown M. S. (1979). Nature.

[cit51] Zaiou M., Arnold K. S., Newhouse Y. M., Innerarity T. L., Weisgraber K. H., Segall M. L., Phillips M. C., Lund-Katz S. (2000). J. Lipid Res..

[cit52] Innerarity T. L., Friedlander E. J., Rall Jr S. C., Weisgraber K. H., Mahley R. W. (1983). J. Biol. Chem..

[cit53] Liu T. W., MacDonald T. D., Shi J., Wilson B. C., Zheng G. (2012). Angew. Chem..

[cit54] LDLR tissue atlas, Human protein atlas, Knut & Alice Wallenberg foundation, http://www.proteinatlas.org/ENSG00000130164-LDLR/tissue, 2017.

[cit55] Bloomer J. R. (1998). J. Gastroenterol. Hepatol..

[cit56] Versluis A. J., Rensen P. C., Rump E. T., Van Berkel T. J., Bijsterbosch M. K. (1998). Br. J. Cancer.

[cit57] Lu C. W., Lo Y. H., Chen C. H., Lin C. Y., Tsai C. H., Chen P. J., Yang Y. F., Wang C. H., Tan C. H., Hou M. F., Yuan S. F. (2017). Cancer Lett..

[cit58] Rotheneder M., Kostner G. M. (1989). Int. J. Cancer.

[cit59] Weisgraber K. H., Innerarity T. L., Mahley R. W. (1978). J. Biol. Chem..

[cit60] Nichols A. V., Krauss R. M., Musliner T. A. (1986). Methods Enzymol..

