# Correction: Tailored theranostic apolipoprotein E3 porphyrin-lipid nanoparticles target glioblastoma

**DOI:** 10.1039/c7sc90047c

**Published:** 2017-07-13

**Authors:** M. A. Rajora, L. Ding, M. Valic, W. Jiang, M. Overchuk, J. Chen, G. Zheng

**Affiliations:** a Princess Margaret Cancer Centre , University Health Network , 101 College Street , Toronto , Ontario M5G 1L7 , Canada . Email: gzheng@uhnresearch.ca; b Institute of Biomaterials and Biomedical Engineering , University of Toronto , 164 College Street , Toronto , Ontario M5S 3G9 , Canada; c Department of Medical Biophysics , University of Toronto , 101 College Street , Toronto , Ontario M5G 1L7 , Canada

## Abstract

Correction for ‘Tailored theranostic apolipoprotein E3 porphyrin-lipid nanoparticles target glioblastoma’ by M. A. Rajora *et al.*, *Chem. Sci.*, 2017, DOI: ; 10.1039/c7sc00732a.



## 

In Fig. 6 of the paper, the labels for the final two sets of treatment groups should be switched around as indicated in the revised figure.

**Fig. 6:**
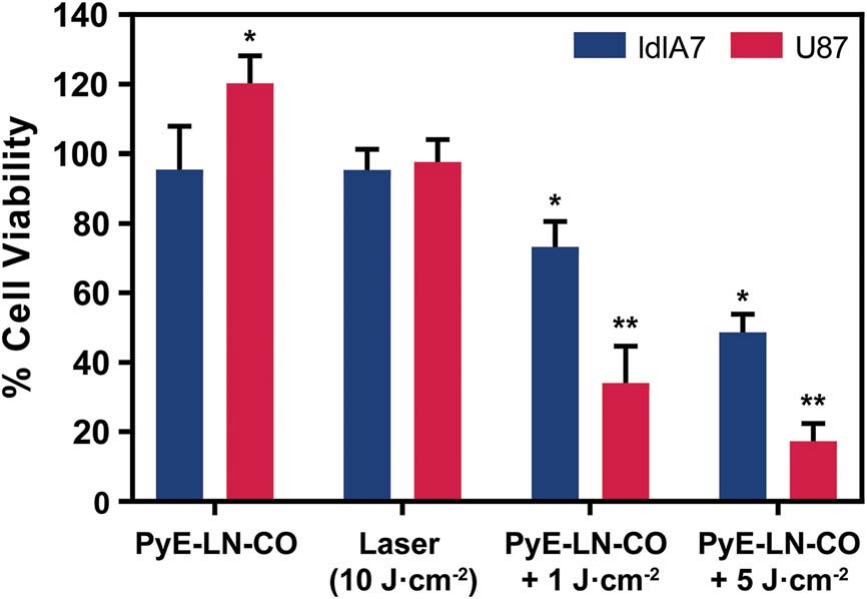
*In vitro* evaluation of pyE-LN PDT sensitization. Cell viability was normalized to untreated cells and is presented as the average of three replicates ± standard deviation. Cells were treated with py-LN-CO (3 μM), laser (671 nm) or a combination of laser and particle. Significant differences (*p < 0.01, *n* = 3) were observed between treated and untreated cells, wherein significantly higher toxicity (**p < 0.01, *n* = 3) was observed in U87 cells *versus* ldlA7 cells treated with particle and laser.

The Royal Society of Chemistry apologises for these errors and any consequent inconvenience to authors and readers.

