# Supplementary material for: Tailored theranostic apolipoprotein E3 porphyrin-lipid nanoparticles target glioblastoma
Source: Chem Sci. 2017 May 23;8(8):5371–84. doi: 10.1039/c7sc00732a (PMC5609152; doi:10.1039/c7sc00732a)
Supplement: Supplementary file 1 [file SC-008-C7SC00732A-s001.pdf]

Electronic Supplementary Material (ESI) for Chemical Science.
This journal is © The Royal Society of Chemistry 2017

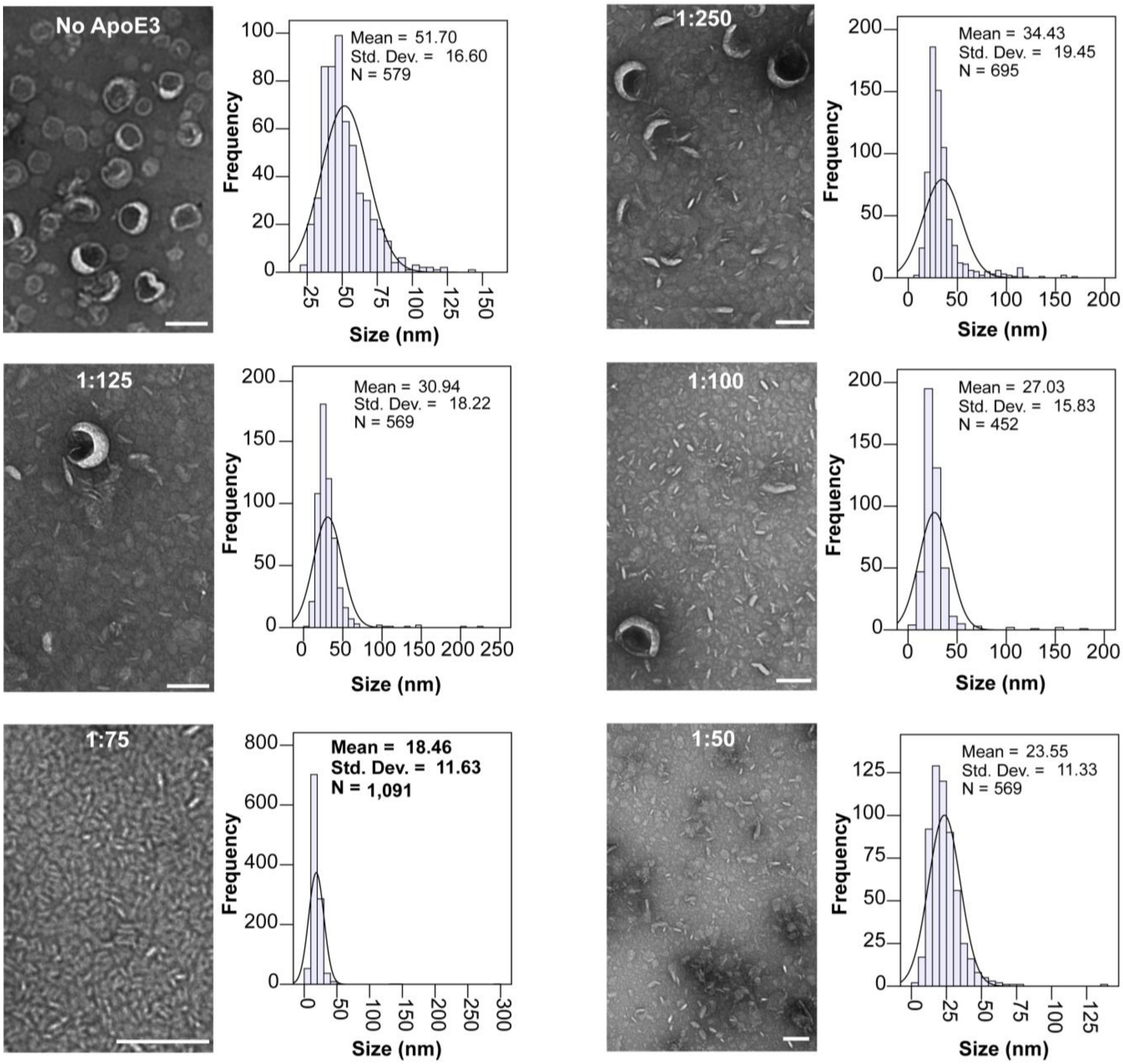

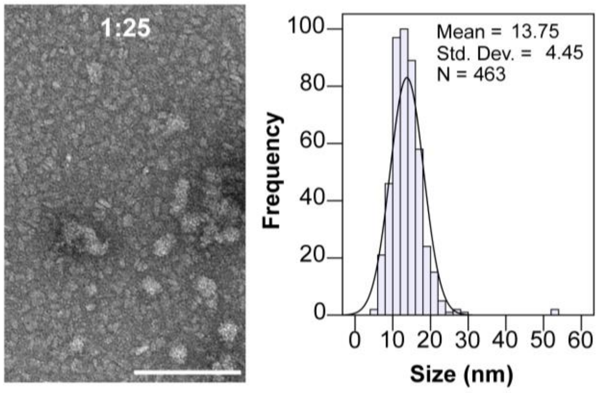

<u>Figure S1:</u> TEM images and size distributions associated with the optimization of the apoE3:lipid molar ratio for the formulation of pyE-LN. ApoE3 was added in increasing quantities to DMPC suspensions to consume vesicles and form discoidal particles. The means provided represent the average size of a minimum of 450 particles (N value indicated) measured using ImageJ from a minimum of three different representative TEM fields of view. A 1:75 apoE3:lipid ratio was selected for further particle formulation. Scale bar = 100 nm.

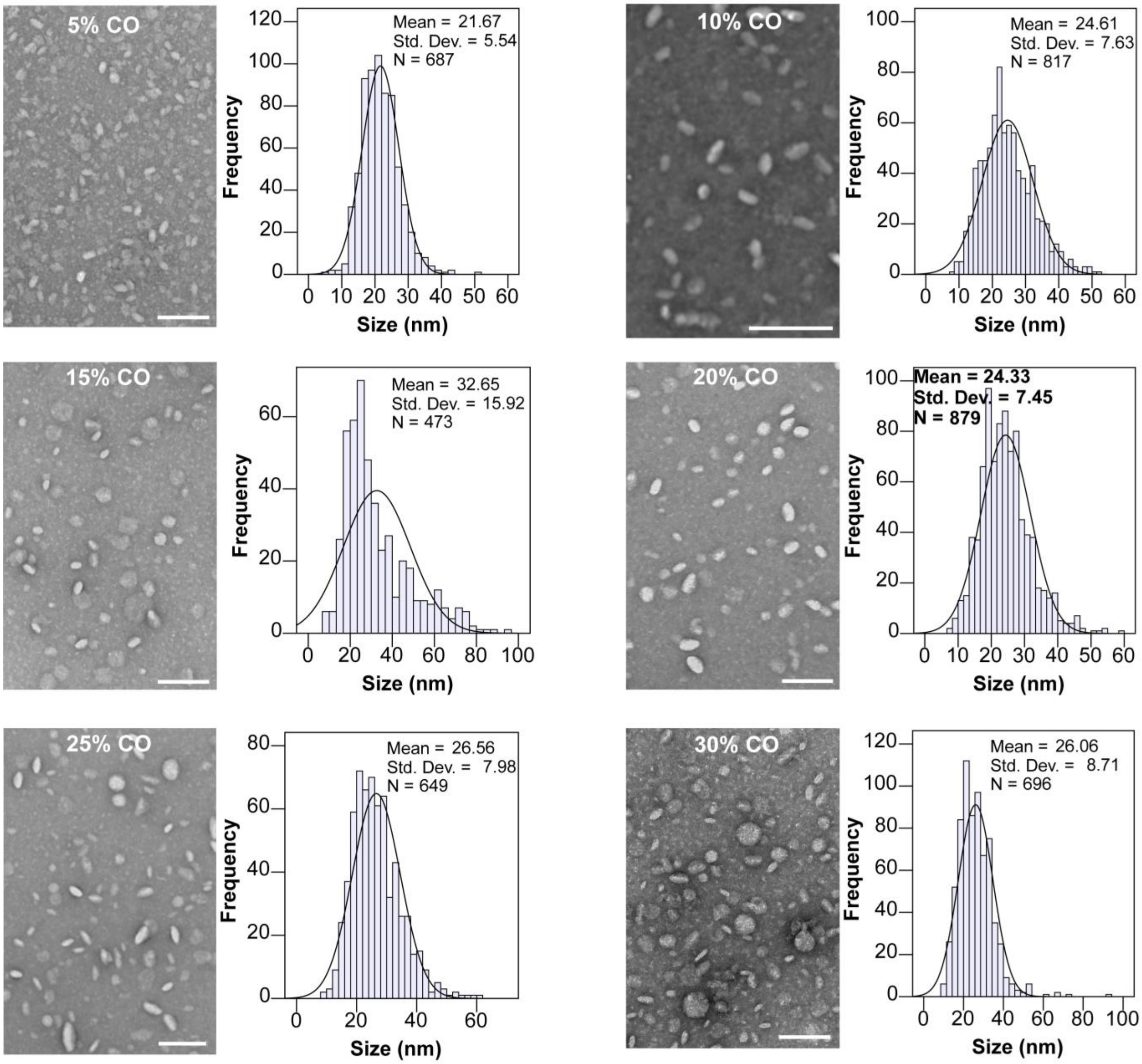

Figure S2: TEM images and size distributions associated with the optimization of the CO/lipid molar ratio, where percentages indicate CO quantity with respect to total moles of lipid. CO was titrated into DMPC lipid films prior to sonication and addition of apoE3 in a 1:75 apoE3:DMPC molar ratio. An increasing addition of CO resulted in a morphological change from discoidal to ellipsoidal and spherical particles, with high CO loading resulting in highly heterogenous particle morphology. The means provided represent the average size of a minimum of 450 particles (N value indicated) measured using ImageJ from a minimum of three different representative TEM fields of view. A loading of 20 molar% CO with respect to total lipid was selected to formulate pyE-LN-CO. Scale bar = 100 nm.

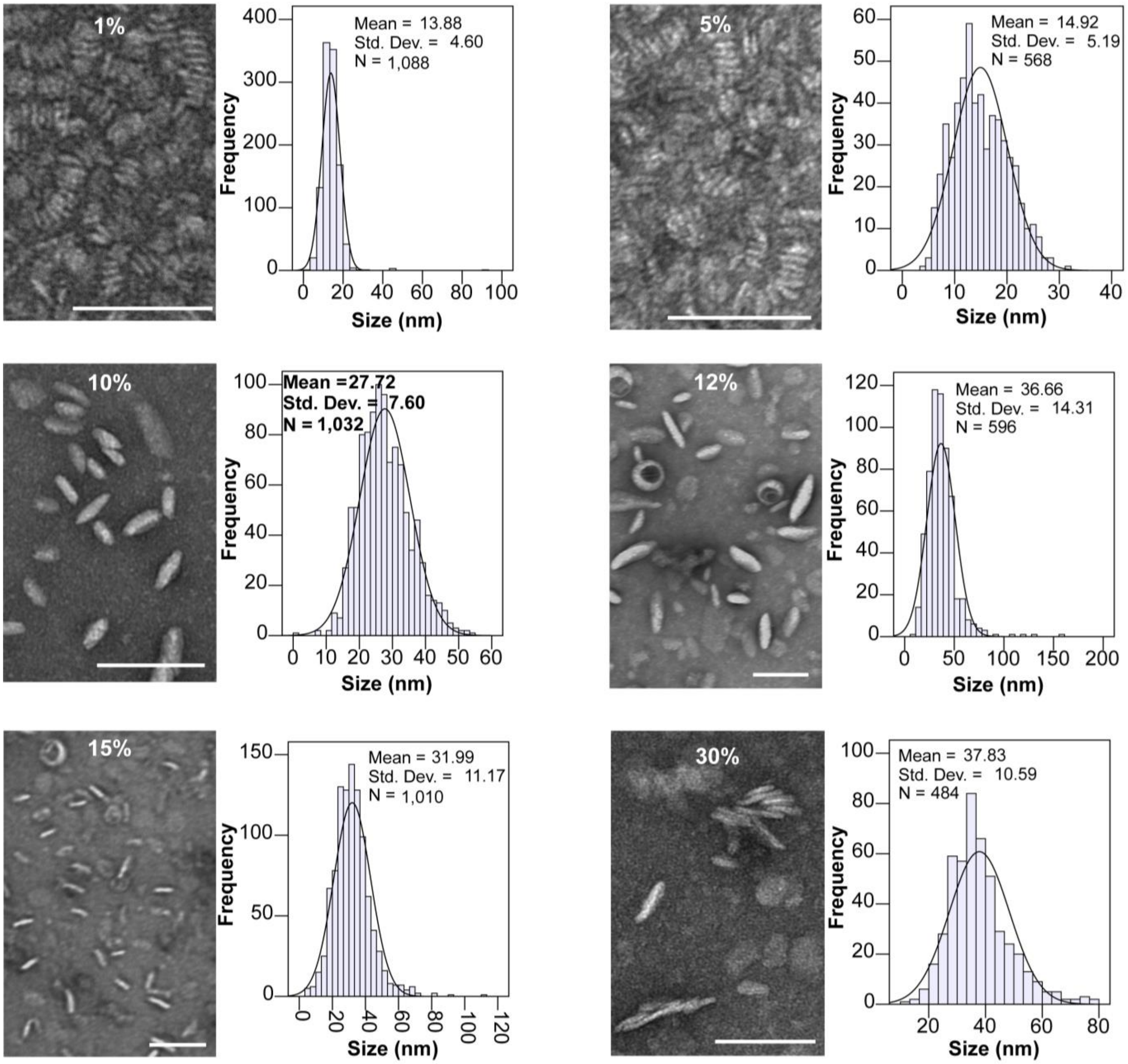

Figure S3: TEM images and size distributions associated with the titration of porphyrin-lipid into pyE-LN-D formulations. Percentages indicate the molar fractions of the total lipid content comprised of porphyrin-lipid. Increasing the porphyrin-lipid fraction increased the mean particle size, which represents the average size of 450 particles (N value indicated) measured using ImageJ from a minimum of three different representative TEM fields of view. A 10% porphyrin-lipid/90% DMPC lipid composition was selected to formulate optimized pyE-LN-D. Scale bar = 100 nm.

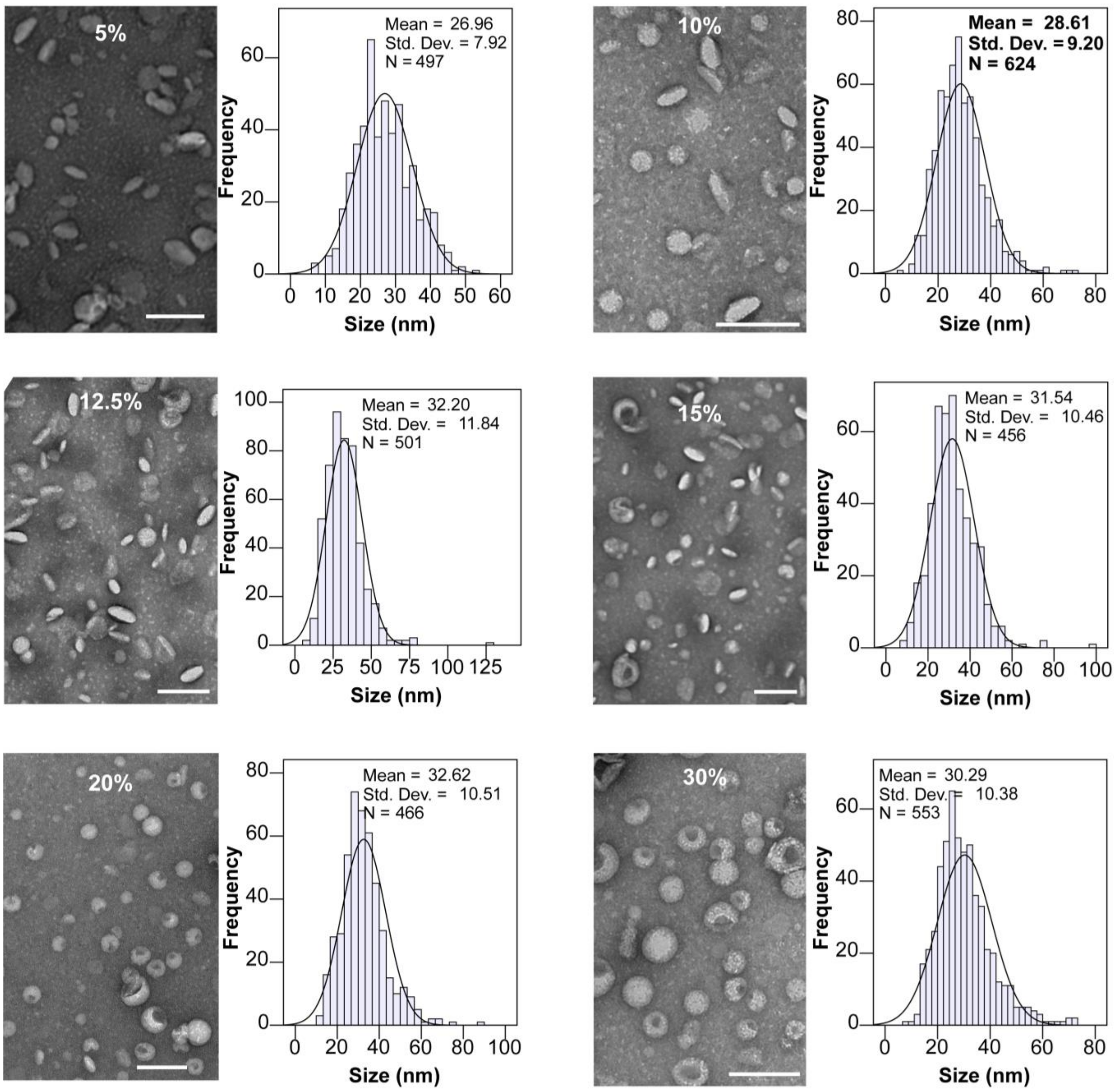

Figure S4: TEM images and size distributions associated with the titration of porphyrin-lipid into pyE-LN-CO formulations. Percentages indicate the molar fractions of the total lipid content comprising porphyrin-lipid. Increasing the porphyrin-lipid fraction increased the number of vesicles formed as observed by TEM. The means provided represent the average size of a minimum of 450 particles (N value indicated) measured using ImageJ from a minimum of three different representative TEM fields of view. A 10% porphyrin-lipid/90% DMPC lipid composition was selected to formulate optimized pyE-LN-D. Scale bar = 100 nm.

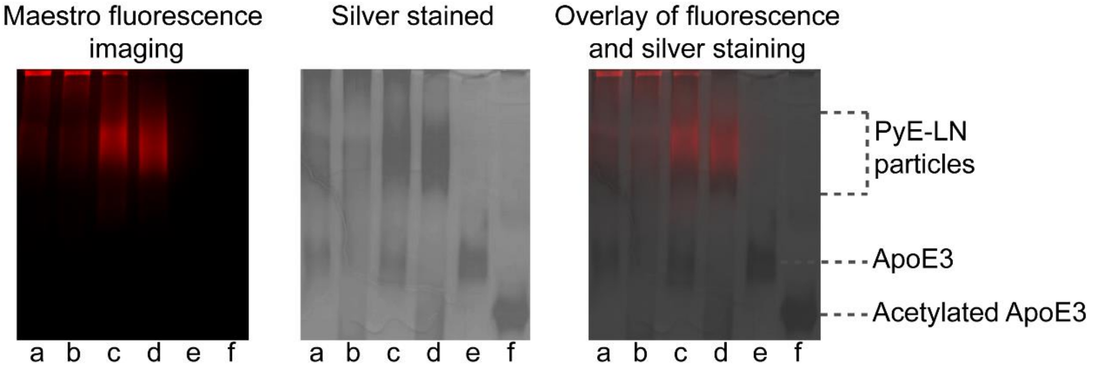

Figure S5: Native polyacrylamide gel electrophoresis of (a) pye-LN-CO, (b) pyE-LN-CO-Ac, (c) pyE-LN-D, (d) pyE-LN-D-Ac, (e) ApoE3, and (f) ApoE3-Ac. The gel was imaged on a CRi Maestro fluorescence imaging system to detect porphyrin signal (616-661 nm excitation, 675 nm longpass emission, 750 ms exposure), subsequent to which the gel was silver stained to visualize apoE3 bands (grey). Image co-registration demonstrates overlap in fluorescence and protein bands suggesting that the porphyrin-lipid and apoE3 in the particle suspensions are co-assembled.

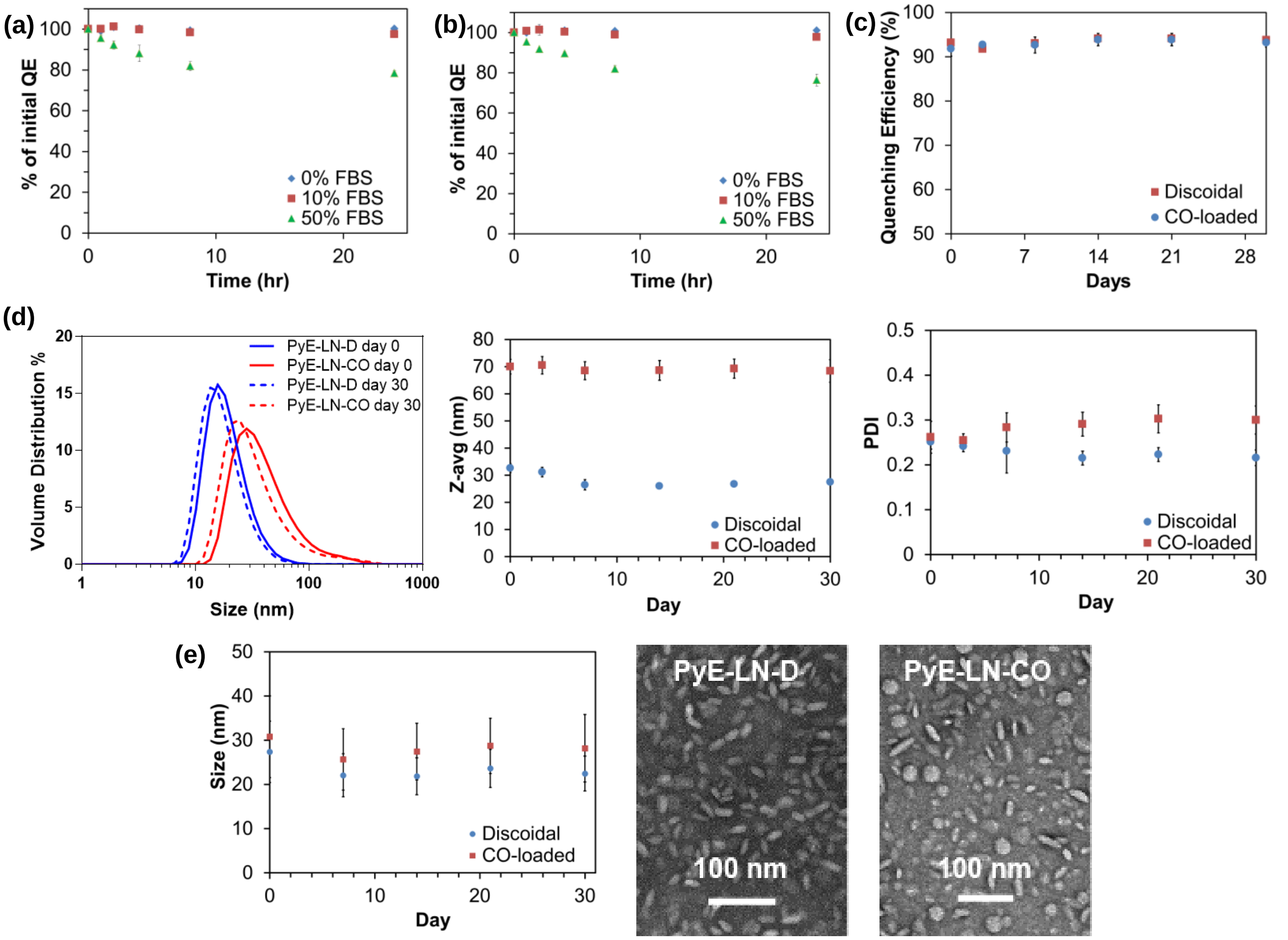

Figure S6: Serum and storage stability of pyE-LN nanoparticles. Serum stability of (a) pyE-LN-D and (b) pyE-LN-CO was evaluated by monitoring the fluorescence quenching efficiency (600-750 nm, 410 nm excitation) of particles dispersed in FBS solutions over a 24 hour period at 37°C. Storage stability of aqueous particle solutions was assessed via quenching efficiency (c), DLS (d), and TEM (e) measurements, conducted on three samples stored at 4°C for 30 days. No significant change (independent samples t-test, 3 samples) in quenching efficiency or increase in particle size (by TEM and DLS) were observed between Day 0 and Day 30. All data points represent the mean of three samples ± standard deviation. TEM size measurements are presented as the average of 450 measurements (150 from each of three samples, measured with ImageJ) ± standard deviation.

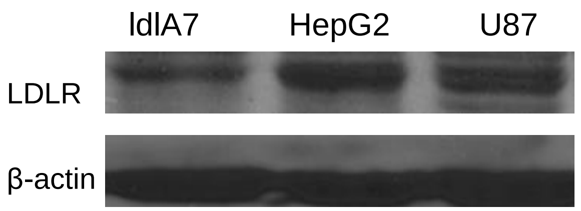

Figure S7: Western blot of ldlA7, U87 and HepG2 cell lysates using anti-LDLR and β-actin antibodies. U87s were found to have high expression of LDLR, similar to that of HepG2 (positive control for LDLR expression), while ldlA7 demonstrated lower LDLR expression.

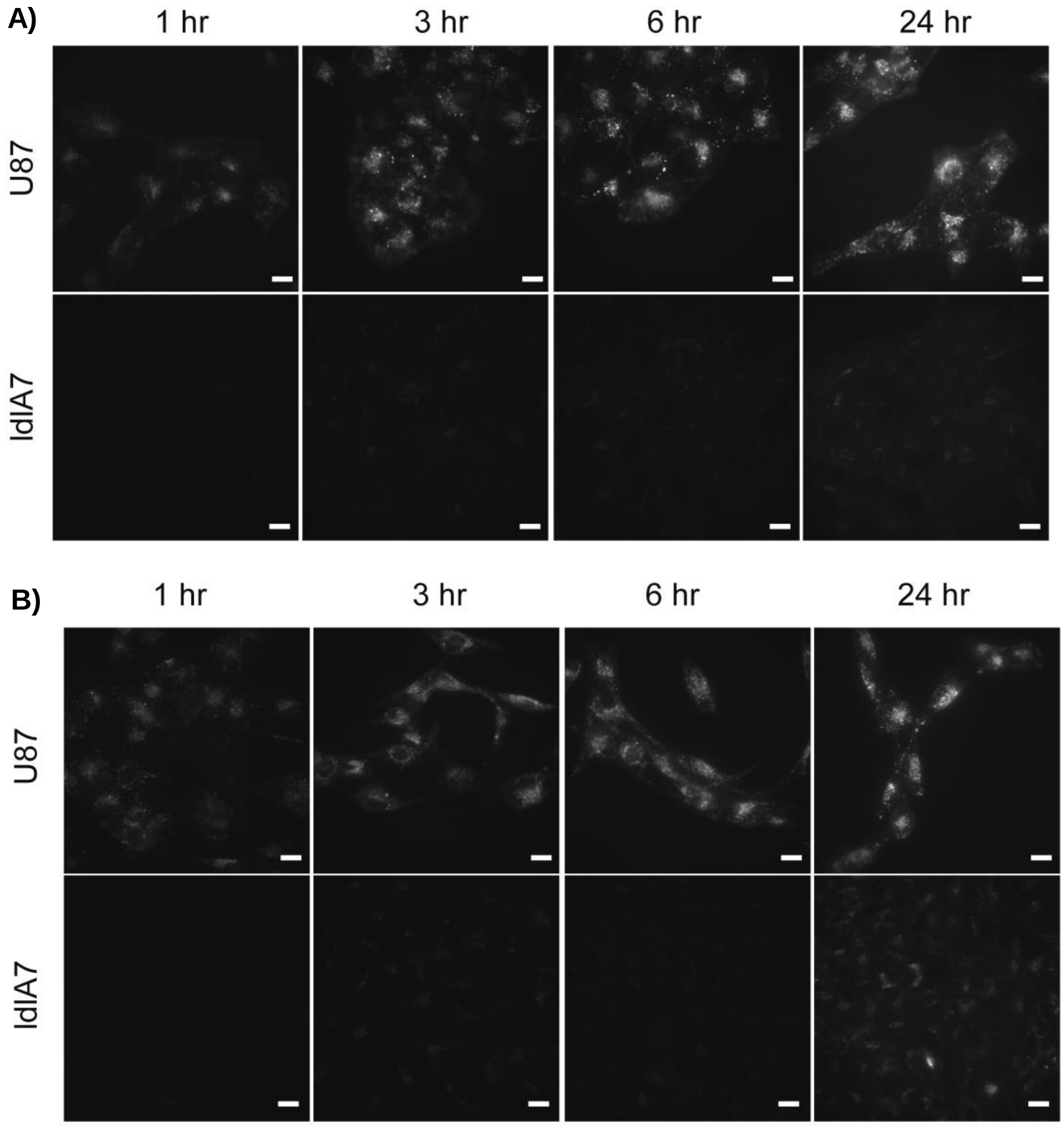

Figure S8: Time-dependent uptake of pyE-LN-D (a) and pyE-LN-CO in U87 (high LDLR expression) and ldlA7 (low LDLR expression) cells. The cells were treated with a porphyrin concentration of 5 uM for 1, 3, 6, or 24 hours prior to washing and imaging via fluorescence microscopy. The porphyrin signal shown above was visualized using a Cy5 filter (628/40 nm excitation, 692/40 nm emission). With equivalent light exposure, higher porphyrin fluorescence signal was observed in the LDLR-expressing U87 cells with increasing treatment times, while fluorescence signal emanating from treated ldlA7 cells was negligibly visible until 24 hours of treatment. Scale bars = 20 µm.

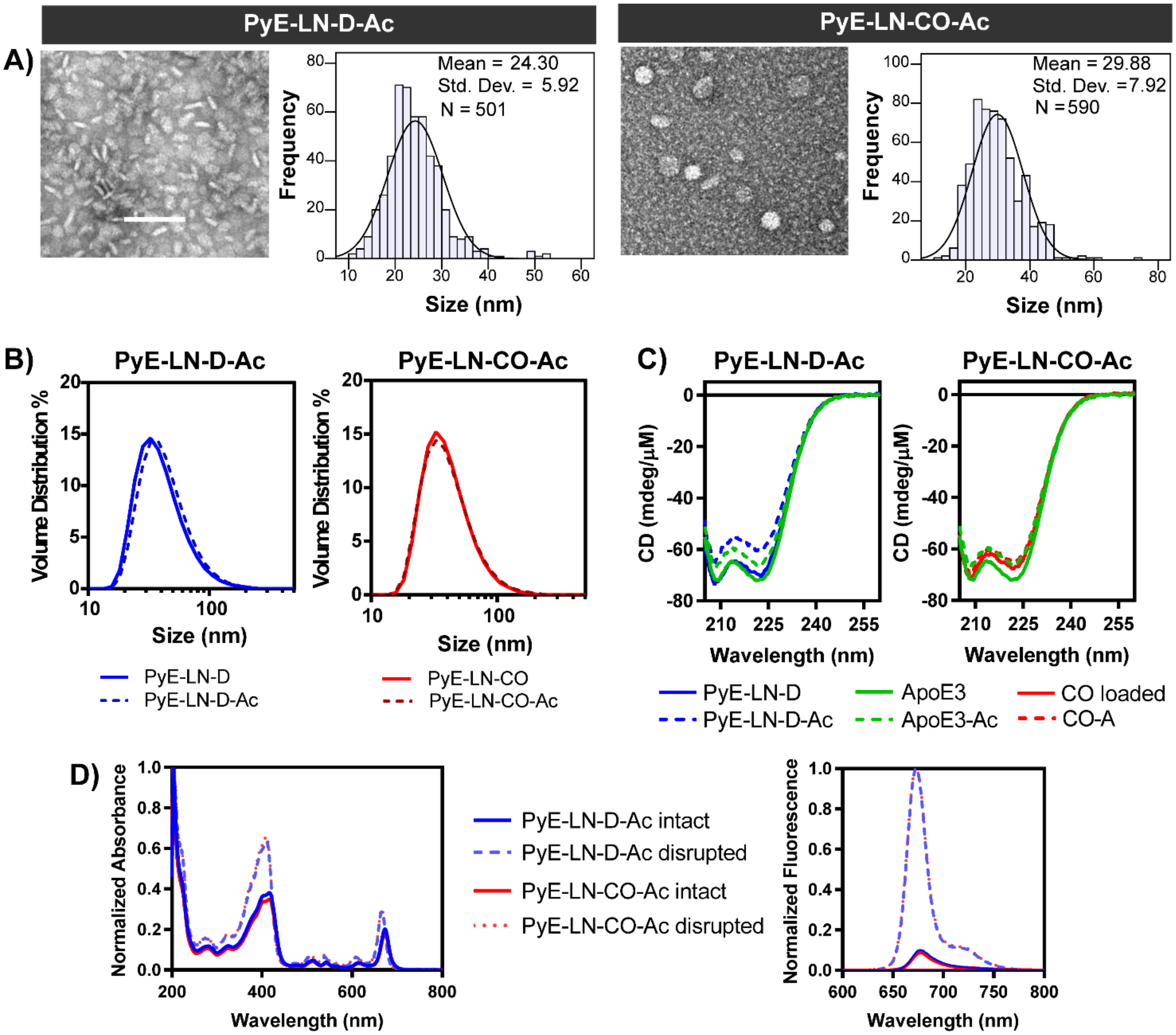

Characterization	PyE-LN-D-Ac	PyE-LN-CO-Ac
Size by TEM (nm)	24 ± 6 (28 ± 8)	30 ± 8 (29 ± 9)
Z-avg (nm)	22.33 (23.71)	61.79 (62.62)
PDI	0.186 (0.183)	0.346 (0.343)
Q_y intact	673 nm (673 nm)	673 nm (673 nm)
Q_y disrupted	666 nm (666 nm)	666 nm (666 nm)
Quenching efficiency (%)	88 (88)	90 (89)

Figure S9: In solution characterization of acetylated discoidal (pyE-LN-D-Ac) and CO-loaded (pyE-LN-CO-Ac) particles via TEM (a), DLS (b), CD (c), and spectrofluorotmetry and photometry (d). Acetylated particles had similar size, CD and optical properties as protein and porphyrin-lipid concentration-equivalent parent pyE-LN particles suspension as summarized in the illustrated table (values in parentheses are associated with the parent pyE-LN particles). Scale bar = 100 nm.

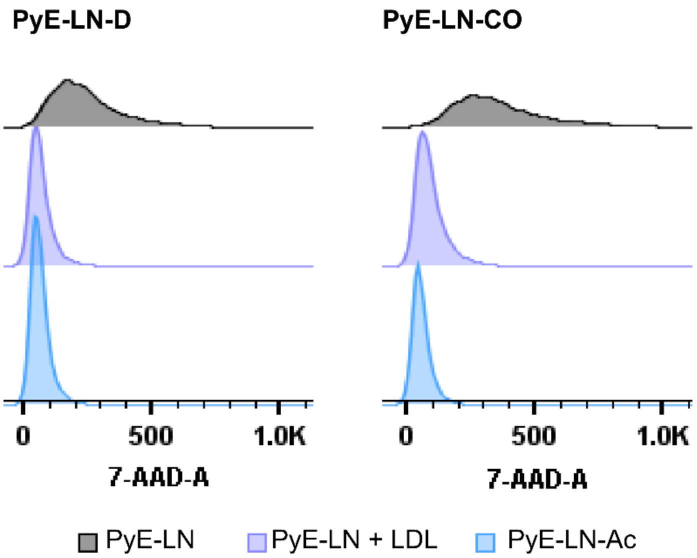

<u>Figure S10:</u> Representative flow cytometry histograms associated with in vitro pyE-LN uptake inhibition in U87 cells.

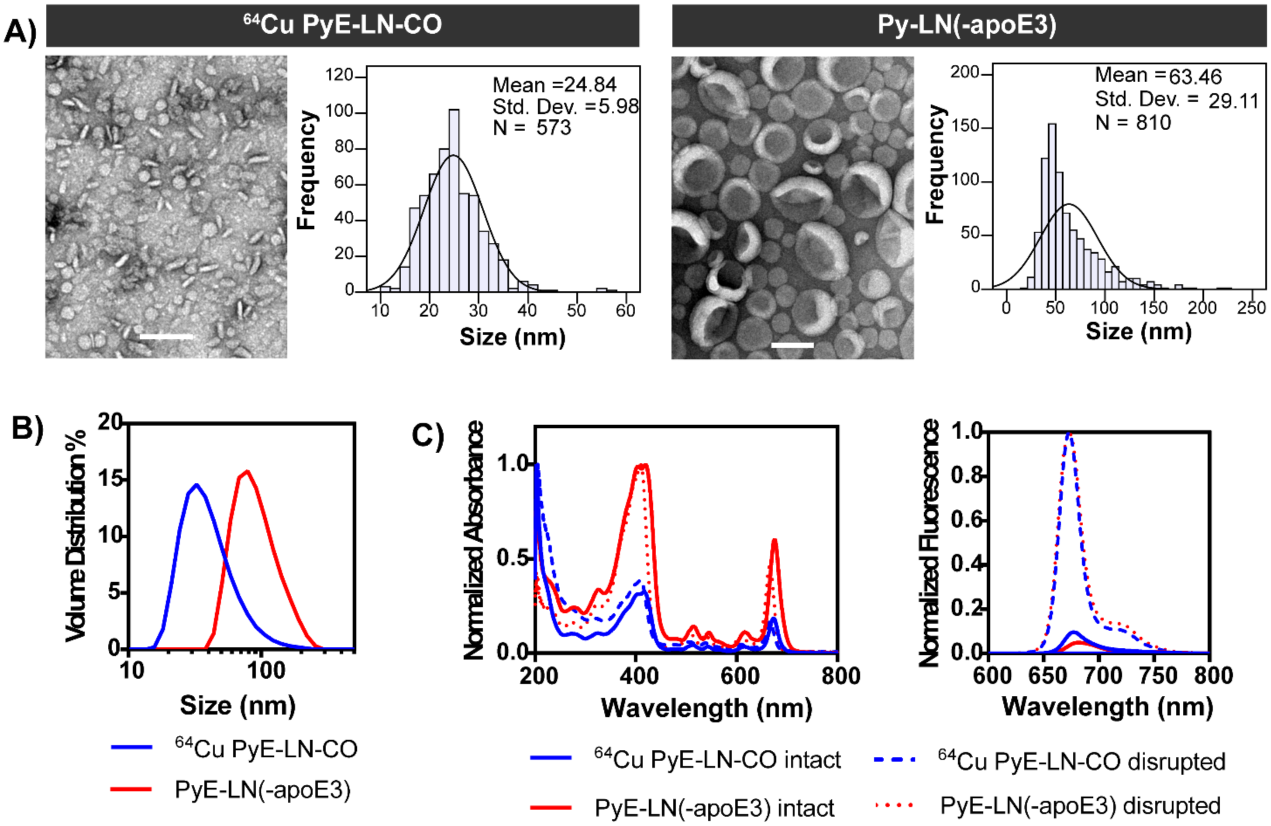

Characterization	^{64}Cu PyE-LN-CO	Py-LN(-apoE3)
Size by TEM (nm)	25 ± 6	69 ± 30
Z-avg (nm)	62.82	99.22
PDI	0.252	0.095
Q_y intact	674	674
Q_y disrupted	666	666
Quenching efficiency (%)	89	94
Radiolabeling efficiency (%)	100 ± 1.6	N/A

Figure S11: Characterization of pyE-LN particles utilized for in vivo studies. ^{64}Cu-PyE-LN-CO particles were allowed to undergo radioactive decay for two weeks prior to characterization alongside apoE3-devoid py-LN(-apoE3) particles via a) TEM, b) DLS, c) spectrofluorometry and spectrophotometry. Scale bar = 100 nm.
